# Human amniotic epithelial cell transplantation improves scar remodeling in a rabbit model of acute vocal fold injury: a pilot study

**DOI:** 10.1186/s13287-022-02701-w

**Published:** 2022-01-25

**Authors:** Yourka D. Tchoukalova, Stephanie R. C. Zacharias, Natalie Mitchell, Cathy Madsen, Cheryl E. Myers, Dina Gadalla, Jessica Skinner, Katarzyna Kopaczka, Roberto Gramignoli, David G. Lott

**Affiliations:** 1 Head and Neck Regenerative Medicine Laboratory, Mayo Clinic Arizona, Scottsdale, AZ USA; 2 Division of Pediatric Otolaryngology, Phoenix Children’s Hospital, Phoenix, AZ USA; 3 Arizona State University, Tempe, AZ USA; 4 Langley Forensic Research Laboratory, Mayo Clinic Arizona, Scottsdale, AZ 85259 USA; 5 Division of Pathology, Department of Laboratory Medicine, Karolinska Institutet, Stockholm, Sweden; 6 Division of Laryngology, Department of Otolaryngology – Head and Neck Surgery, Mayo Clinic Arizona, 5777 East Mayo Boulevard, Phoenix, AZ 85054 USA

**Keywords:** Vocal fold, Wound healing, Amnionic epithelial cells, Extracellular matrix, Proteomics, Regenerative medicine, Tissue engineering, Fibrosis

## Abstract

**Objective:**

To gain insight into the molecular mechanisms underlying the early stages of vocal fold extracellular matrix (ECM) remodeling after a mid-membranous injury resulting from the use of human amniotic epithelial cells (hAEC), as a novel regenerative medicine cell-based therapy.

**Methods:**

Vocal folds of six female, New Zealand White rabbits were bilaterally injured. Three rabbits had immediate bilateral direct injection of 1 × 10^6^ hAEC in 100 µl of saline solution (hAEC) and three with 100 µl of saline solution (controls, CTR). Rabbits were euthanized 6 weeks after injury. Proteomic analyses (in-gel trypsin protein digestion, LC–MS/MS, protein identification using Proteome Discoverer and the Uniprot *Oryctolagus cuniculus* (Rabbit) proteome) and histological analyses were performed.

**Results:**

hAEC treatment significantly increased the expression of ECM proteins, elastin microfibril interface-located protein 1 (EMILIN-1) and myocilin that are primarily involved in elastogenesis of blood vessels and granulation tissue. A reactome pathway analysis showed increased activity of the anchoring fibril formation by collagen I and laminin, providing mechanical stability and activation of cell signaling pathways regulating cell function. hAEC increased the abundance of keratin 1 indicating accelerated induction of the differentiation programming of the basal epithelial cells and, thereby, improved barrier function. Lastly, upregulation of Rab GDP dissociation inhibitor indicates that hAEC activate the vesicle endocytic and exocytic pathways, supporting the exosome-mediated activation of cell–matrix and cell-to-cell interactions.

**Conclusions:**

This pilot study suggests that injection of hAEC into an injured rabbit vocal fold favorably alters ECM composition creating a microenvironment that accelerates differentiation of regenerated epithelium and promotes stabilization of new blood vessels indicative of accelerated and improved repair.

**Supplementary Information:**

The online version contains supplementary material available at 10.1186/s13287-022-02701-w.

## Background

Vocal fold trauma is a relatively frequent event caused by mechanical injury, prolonged inflammation, radiotherapy, or surgery. A common outcome from the healing process of vocal fold injury is fibrosis or scarring, which changes the viscoelasticity of the vocal fold, leading to dysphonia: poor voice quality, hoarseness, loss of vocal range, and strain [1]. Dysphonia is known to cause decreased quality of life, time off work, job loss, and decreased social and emotional well-being [2].

The vocal fold consists of three main layers: the layer of epithelial cells with non-keratinized stratified squamous phenotype on the luminal side, which forms the barrier with the external environment; the underlying lamina propria, which provides strength and elasticity/compliance, and the deep vocalis muscle. Upon injury of the vocal fold, the epithelial layer is restored by migration and proliferation of epithelial cells that are adjacent to the wound site (reepithelization). This leads to wound closure which is followed by epithelial differentiation and stratification to establish barrier function [3]. Lamina propria is restored by development of granulation tissue involving differentiation of fibroblasts into extracellular matrix (ECM) producing contractile myofibroblasts coupled with revascularization [4]. There is a continuous bidirectional collaboration between fibroblasts, epithelial, endothelial, immune, and other cell types, as well as between cells and their surrounding ECM molecules, termed dynamic reciprocity. This involves regulation of cell function by biochemical and mechanical signals from ECM molecules through specific receptor-ligand interactions, which in turn influences the composition and quantity of the ECM produced by the cells [4]. In general, the healing process encompasses three sequential and overlapping phases: inflammation, tissue formation/cell proliferation, and ECM remodeling. Timely resolution of the initial inflammatory process and wound closure is critical to prevent excessive disordered deposition and subsequent inadequate remodeling of ECM in the lamina propria (fibrosis), thereby preventing stiffness and impaired vibratory properties.

Current standard of care is steroid injection by itself or in combination with vocal fold surgery to reduce inflammation; however, this has limited benefit [5]. During the past years, regenerative medicine and tissue engineering approaches including local injection of acellular matrix or purified components and autologous or allogeneic mesenchymal stem cell (MSC) transplantation have shown an encouraging level of efficacy in reduction of inflammatory outbreak, followed by promising fibrosis control [6–8]. Such important capacity to regulate the microenvironment and enhance matrix remodeling envisages restoration of lamina propria structure and vocal function [9, 10]. Nevertheless, these adult MSC-based therapies have not yet become widely used in clinical practice. A survey of 131 clinicians, scientists, and industry experts identified availability and manufacturing as key barriers to the adoption of cellular therapies; with efficacy, regulation, and cost effectiveness as other roadblocks to a wide and established medical treatments [11].

Use of human amniotic epithelial cells (hAEC) has recently emerged as a new cellular therapy with unlimited supply and regenerative potential. The amniotic membrane is a tissue of fetal origin, not maternal, originating from the epiblast during gastrulation before three germ layers are formed. The hAEC form a unicellular layer in the innermost membrane of the amniotic sac and can be easily isolated at the end of pregnancy with no additional risks or impediment for the delivery of the baby. They maintain multipotency characteristics and the expression of surface markers and genes commonly observed in embryonic stem cells [12]. Transplantation of primary hAEC has been proven safe [12] and cell quality has been confirmed upon cryogenic preservation [13]. hAEC isolated from full-term human placentae have been preclinically evaluated in different settings, where local injection or administration by intravenous infusion has been reported [14], highlighting hAEC capacity to engraft and survive upon allogeneic and xenogeneic transplantation in immune-competent animals or patients without immunosuppression support [15, 16]. Initially, the corrective and supportive effects offered by primary hAEC upon transplantation of organs with secretory functions such as liver or lungs have been validated and documented [15, 17]. Further investigations found that hAEC transplantation can improve the dysfunction of other organs and the healing of acute and chronic tissue damage. Further investigations found that hAEC transplantation can improve the dysfunction of other organs and the healing of acute and chronic tissue damage. Remarkably, hAEC injection not only prevents acute pro-inflammatory injury, modulates recipient immune-response, and inhibits recognition and rejection, but also reverses established fibrosis [18, 19]. Additionally, hAEC-derived paracrine mediators have been reported to reduce liver fibrosis [20]. The beneficial healing effects of hAEC on full-thickness dermal wounds are comparable in efficiency to human MSC [21]. However, the efficacy of hAEC in the treatment of injured vocal folds has never been tested before. We used a rabbit xenogeneic transplantation model and evaluated the effect of hAEC on the protein profiles and functional protein pathways 6 weeks after vocal fold injury. There are many published studies using various cell therapies for vocal fold injury in rabbits. The interval between the injury and the evaluation varies widely between 4 weeks and 12 months [9]. Among these studies, significant improvement of the healing is reported as early as 4 weeks and two studies found beneficial effects at 6 weeks after injection of adipose derived stem cells [22, 23].

## Materials and methods

### Preparation of human amniotic epithelial cells

#### Human AEC isolation

Three human placentae were procured at Karolinska Institute Hospital (Stockholm, Sweden) from uncomplicated full-term cesarean section (ethical permit number: 2015/419-34/4). Signed, informed consent and serological analysis were collected and adequate to proceed with cell isolation. Intact placenta was delivered to the laboratory and processed within 3 h post-partum following the protocol previously described [24]. Briefly, the amnion membrane was mechanically removed and washed extensively to remove blood. The amnion membrane was incubated with TrypLE 10× (ThermoFisher, Grand Island, NY) for 30 min at 37 °C to specifically release epithelial cells. The dispersed hAEC were collected by centrifugation and filtered through a 100 µm cell strainer. Resulting hAEC were immediately frozen as previously described [13]. Cell viability after isolation and thawing was determined by the Trypan Blue exclusion method (ThermoFisher, Waltham, MA).

#### Flow cytometry analysis

Cellular fraction extracted from the placenta was analyzed by flow cytometry to determine purity of the epithelial population prior to use for injection. The following surface markers were used: CD326 (clone HEA-125, Miltenyi Biotech), CD49f (clone GoH3, BD Biosciences, San Jose, CA-USA) [hAEC], CD49d (clone, BD), CD105 (clone SN6, BD)[stromal cells], CD45 (clone T29/33, DAKO) [hematopoietic cells], and HLA-ABC (clone DX17, BD), HLA-G (clone MEM-G/9, ExBio Praha, a.s., Czech Republic) and HLA-DR (clone G46-6, BD) [HLA class Ia, Ib, and II, respectively]) as previously described [13].

#### Preparation of cells for injection

Shortly before application in the treatment group, cells from the three donors were thawed in DMEM supplemented with 1% human serum albumin. After centrifugation (400*g* for 5 min), cell pellets were reconstituted in HBSS (Gibco, Gaithersburg, MD), counted, and equal numbers of cells from each donor were pooled together. Cell suspensions were centrifuged (400*g* for 5 min) again and reconstituted in physiological saline (Baxter, Deerfield, IL) to achieve a concentration of 1 × 10^7^ cells/ml. A hundred microliters of cell suspension containing 1 × 10^6^ hAEC were injected in each vocal fold of the treated rabbits (*n* = 3) and 100 µl of saline was injected into the CTR rabbits (*n* = 3).

### Animal surgery

This study was approved by the Institutional Animal Care and Use Committee of Mayo Clinic. Six female New Zealand White rabbits (Charles River, Hollister CA; 2.5 kg) received buprenorphine SR (0.18 mg/kg SQ; ZooPharm™ Formulation, Windsor, CO) prior to surgery. Anesthesia was induced by using ketamine hydrochloride (35 mg/kg IM; Ketaset®, 100 mg ketamine/ml, Wyeth/Fort Dodge Animal Health, Overland Park, KS), xylazine hydrochloride (5 mg/kg IM; AnaSed®, 100 mg xylazine/ml, Lloyd, Shenandoah, IA), and acepromazine maleate (0.75 mg/kg IM; VetOne®, 10 mg acepromazine/ml, Boise, ID). Vocal fold injury was conducted under visual guidance of a pediatric endoscope and a video monitor. Once visualized, 3 mm cupped forceps were used to create a defect in the mid-membranous vocal folds bilaterally. The defect extended to the superficial portion of the thyroarytenoid muscle. Great care was taken to ensure the length and depth of the injury was the same in all vocal folds. Immediately post-injury, the vocal folds were injected bilaterally into the center of the thyroarytenoid muscle injury using a Kleinsasser laryngeal injection needle with either 1 × 10^6^ hAEC in 100 µl of saline solution (hAEC, *n* = 3 pairs of vocal folds) or with 100 µl of saline (CTR, *n* = 3 pairs vocal folds). This dose was chosen based on evidences of the efficacy and safety of the injections of 1 × 10^6^ AECs in several different animal models for different application [15, 17] and of administering the same cell dose (per kg weight) in a phase I clinical trial, where hAEC has been injected in prenatal babies to support regeneration and activity in the upper airways [16]. Additionally, 1 million cell dose has been reported in rabbit vocal fold damage preclinical model, where mesenchymal stem cells have shown to reduce inflammation and scar formation in vocal folds [22, 25]. No complications were reported with recovery. All rabbits were humanely euthanized with a pentobarbital sodium and phenytoin overdose (3 ml IV; Euthasol®, 390 mg pentobarbital sodium, 50 mg phenytoin sodium/ml, Virbac AH, Inc. Fort Worth, TX) after 6 weeks to evaluate wound healing responses during the early fibrotic ECM remodeling phase of scar maturation.

### Vocal fold excision

After euthanasia, a total laryngectomy was performed, and the larynges were bisected between the arytenoids. In all animals, the left hemilarynx was fixed in neutral buffered formalin and embedded in paraffin for subsequent histological analysis of the injured vocal fold. The right vocal fold was excised and snap-frozen in liquid nitrogen and stored at − 80 °C until extraction of the proteins.

### Mass spectrometry

#### Protein isolation and quantification

Proteins in the tissue samples were extracted using AllPrep® DNA/RNA/Protein Mini Kit (Qiagen, Hilden, Germany) following manufacturer’s protocols. Protein concentrations were measured using Pierce® BCA protein assay kit (ThermoFisher Sci.).

#### In-gel protein digestion

In-gel trypsin digestion protocols were performed as previously described [26]. Briefly, proteins were reduced in 10 mM DTT (GBiosciences, St. Louis, MO) for 30 min at 60 °C, and then alkylated with 55 mM iodoacetamide (IAA, MilliporeSigma, St. Louis, MO) for 30 min at room temperature in the dark, prior to 37 °C overnight digestion with Pierce™ MS Grade trypsin protease (Thermo) diluted to 20 ng/ml in 100 mM ammonium bicarbonate (MilliporeSigma). The peptides were then dried down using CentriVap DNA vacuum concentrator (Labconco, Kansas City, MO) and stored at − 80 °C until LC–MS analysis.

#### Proteomic mass spectrometry

Protein digests were reconstituted in 0.1% formic acid (ThermoFisher Sci.) and analyzed using LC–MS/MS by loading onto a Dionex UltiMate® 3000 RSLC liquid chromatography (LC) system (ThermoFisher Sci.) using a PepMap RSLC C18 2um, 75 um × 50 cm EASY-Spray™ column (ThermoFisher Sci.). Peptides were separated using a 0.3 uL/min LC gradient comprising 2–90% mobile phase B in 0–150 min. Mobile phase A was 0.1% formic acid in Milli Q water and mobile phase B was 0.1% FA in acetonitrile (MilliporeSigma). Eluting peptides were directly injected into an Orbitrap Elite Velos mass spectrometer (ThermoFisher Sci.) and ionized using collision-induced dissociation (CID) in positive ion mode. A “top 15” data-dependent MS/MS analysis was performed (acquisition of a full scan spectrum followed by CID mass spectra of the 15 most abundant ions in the survey scan). The data was submitted to the MassIVE repository: ftp://massive.ucsd.edu/MSV000087132/ for public access.

#### Protein identification and label-free protein quantification

Database searching was performed using Sequest (ThermoFisher Sci.) in Proteome Discoverer v1.4.1.14 (Thermo) against reference FASTA proteomes of the Uniprot *Oryctolagus cuniculus* (Rabbit) proteome (UP000001811, December 13, 2018 release). Searches were performed using a fragment tolerance of 0.60 Da (monoisotopic), parent tolerance of 10 ppm (monoisotopic), with carbamidomethyl of cysteine as a fixed and oxidation of methionine as variable modifications and maximum missed cleavages allowed of up to 2. Protein identifications were accepted if they achieved a minimum of 2 peptides per protein and a false discovery rate (FDR) of < 1%. Label-free protein quantification was performed using normalization of spectral abundance factors (NSAF) in Scaffold (v4.8.7, Proteome Software Inc.).

### Histology and immunohistochemistry

The excised vocal folds were fixed in neutral buffered 10% formalin (Cardinal Health, Waukegan, IL) and embedded in paraffin. Sections (7 μm) were de-paraffinized and rehydrated, then stained with hematoxylin and eosin (H&E) to evaluate gross histology. Masson’s trichrome and picrosirius red (the latter examined under polarized light) were used to visualize connective tissue and collagen, respectively. Tissues were stained with periodic acid–Schiff (PAS) to see mucosubstances in mucus-secreting cells.

Immunohistochemical staining was performed using antibodies against the proliferation marker Ki67 (Novus Biologicals LLC, Centennial CO; #NBP2-22112, 1:100) and the Vectastain Elite ABC avidin biotin kit (Vector Laboratories, Burlingame, CA; cat# PK-6102) following the manufacturer’s instructions. Antigen retrieval was done with citrate-based pH 6 buffer (Agilent Dako, Santa Clara, CA) under pressure for 10 min at 110 °C. The Ki67 positive cells were visualized with 3,3′-diaminobenzidine (DAB) (Vector Laboratories). Sections were counterstained with Mayer’s Hematoxylin–Lillie’s Modification (Volu-Sol, Salt Lake City, UT). Positive and negative tissue controls were included for all stains.

Immunofluorescent staining was performed using mouse monoclonal antibodies against the myofibroblast marker smooth muscle alpha actin (αSMA) (Nordic Biosite, Wayne, PA; #BSH 7459, 1:200), the endothelial marker CD31 (clone JC/70A, # V3143, NSJ bioreagents, San Diego CA; 1:200), and myocilin (Proteintech, Rosemont IL, #60357-1, clone 4B1F6) to detect its expression in rabbit vocal fold and other laryngeal and tracheal structures. Specifically, the expression of myocilin has not been previously reported in vocal folds in animals and humans. To understand whether this is a species-dependent feature, we additionally stained one human vocal fold. The larynx of a deceased 14-year-old male was excised by a pathologist at a Phoenix Children’s Hospital after obtaining an approval of a Decedent Research Request by its Institutional Review Board. The larynx was placed in formalin and transported to Mayo Clinic where a hemilaryngectomy was completed and the vocal fold was dissected and embedded in paraffin. Antigen retrieval was done with either citrate-based pH 6 buffer (Agilent Dako, Santa Clara, CA) under pressure for 15 min at 110 °C for αSMA and CD31 or Tris–EDTA buffer (pH 9) (Sigma, St. Louis, MO) under pressure for 5 min at 110 °C for myocilin. Serum-free protein block (Agilent-DAKO, Santa Clara, CA) supplemented with 0.1% saponin (Sigma) was used to block and permeabilize. Tissues were incubated with the primary antibodies for 1 h at room temperature followed by 30 min in secondary goat anti mouse Alexa Fluor 546 IgG (Invitrogen, Carlsbad, CA). The antibodies were diluted 1:200 times in antibody diluent with background reducing components (Agilent-DAKO) and 0.1% saponin. Sections were cover-slipped using ProLong Gold mounting medium with DAPI (Life Technologies. Carlsbad CA, product id: #P36931). Images were taken using Olympus IX83 inverted microscope and EVOS M5000 imaging system (ThermoFisher Sci.).

### Data analysis

#### Protein expression

The differences in protein abundance between hAEC and CTR groups for each time point were determined by analysis of variation (ANOVA) statistical tests performed in Scaffold (v4.8.7, Proteome Software Inc., Portland, OR) followed by an adjustment for the multiplicity of tests. *p* < 0.05 was deemed significant.

#### Pathway analysis

To determine how the differentially expressed proteins affect cellular function, the differentially expressed proteins with non-adjusted *p* < 0.05 were submitted for a pathways overrepresentation (enrichment) analysis (www.reactome.com). This program evaluates the submitted list against every pathway from the reactome knowledgebase for *Homo sapiens* where orthologous reactions for rabbits are available. A binomial test is used to calculate the probability shown for each result, and the *p* values are corrected for the multiple testing (Benjamini–Hochberg procedure) [27].

#### Immunohistochemical and immunofluorescent analyses

We determined the percent Ki67+ in the immunohistochemical staining by counting each the diaminobenzidine (DAB)-stained cells and the total number of hematoxylin-positive nuclei in the same area of the stromal or mucosal regions of the AEC-treated and control vocal folds tissues using ImageJ-win64 (Fiji) software and calculating the DAB/hematoxylin ratio. To further evaluate the granulation tissue developed in the lamina propria, we performed immunofluorescent staining for αSMA, which is expressed by both mural vascular cells and fibroblasts, and for CD31 to visualize separately solely the blood vessels. Of note, the blood vessels in the vocal fold are small including arterioles, venules, and capillaries. They all are surrounded by pericytes providing a unique support to withstand the mechanical forces of phonation [28]. Therefore, we subtracted the number of blood vessels identified by CD31+ cells from the number of structures formed by αSMA+ cells to calculate the number of fibroblasts. The quantification of new vessels within the aSMA and CD31 stained identical sections was done using ArcGIS, a software that enables the user to store and manipulate spatial data [29]. Spatial data in ArcGIS is usually analyzed in reference to the surface of the earth, a set of data points that biological micrographs lack. Thus, micrographs were projected into ArcGIS using an appropriate coordinate system according to recent methods presented by Michener et al. [30]. Due to the one-dimensional aspect of histological imaging, the Universal Web Mercator projection, which creates minimal distortion of flat objects, was used [31]. An original micrograph with the scale bar embedded was imported as a separate layer and used to verify the scale established by the ArcGIS Pro coordinate system [30, 32, 33]. To generate vector features, each slide was imported as a raster image into the ArcMap interface of ArcGIS (ArcGIS v.10.3 ESRI) and analyzed using the ArcScan extension and auto outline vectorization tool. Auto vectorization is a technique for converting raster data into vector polygons using adjacent raster cells to create borders representative of tissue features [32]. Optimal image resolution and noise reduction facilitated by CD31 and α-SMA staining as well as basic level adjustments of brightness and contrast were used to enable a successful vectorization. The number of fibroblasts was estimated as descried above. We also calculated the density (blood vessels count per area) and average lumen area (the ratio of the sum of the lumen area of blood vessels to the count within the same area). The difference in the means between the groups was tested using *t* test and considered significant if *p* value was 0.05 or less.

## Results

Cell viability measured after the cryogenic preservation of hAEC was 95 + 3%. The presence of surface proteins characteristic for epithelial cells was evaluated to confirm cell suspension homogeneity: All three hAEC preparations were mostly positive for epithelial markers (CD326 and CD49f) and negative for MSC markers as CD49d, CD90 or CD105 (data not shown).

All the animals tolerated transplant procedures with no signs of stress or damage due to local injection.

### Mass spectrometry

**Fig. 1:**
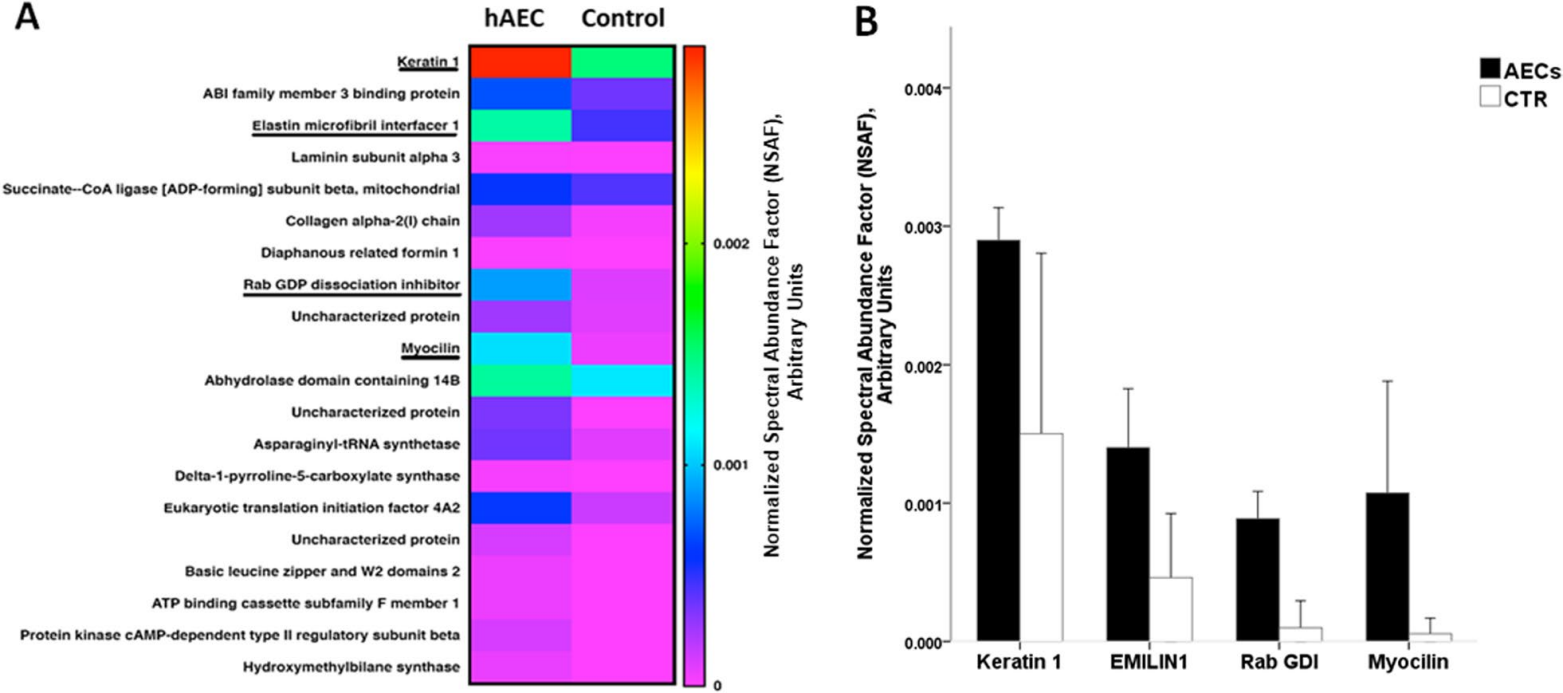
Significantly more abundant proteins in hAEC compared to CTR. A heat map including proteins with non-adjusted *p* < 0.05 (**A**) and adjusted *p* < 0.05 (**A**, underlined). The latter are plotted in a bar graph (**B**)

The list of identified proteins in Fig. 1 shows the proteins with higher abundance in the hAEC-treated group compared to the CTR group. From the group of upregulated proteins with non-adjusted *p* < 0.05, shown in the heat map (Fig. 1A), four maintained their significance after adjustment for multiple testing: keratin 1, elastin microfibril interface-located protein 1 (EMILIN), Rab GDP dissociation inhibitor (GDI), and myocilin (Fig. 1A underlined and B). We found only one protein, Ig kappa-b4 chain C region, which was significantly (adjusted *p* < 0.05) less abundant in hAEC versus CTR.

### Pathway analysis

The enriched pathways are given in the original reactome report provided as an Additional file 1. This analysis presents a genome-wide overview of the hierarchical pathway arrangement demonstrating that the top-level pathways fall into the categories of ECM biology, with the most significant pathway (FDR 0.027) being the anchoring fibril formation involving collagen I and laminin. A selection of the enriched pathways identified by collagen I and laminin is presented in Table 1. Notably, it includes enrichment of met-signaling pathways (FDR 0.059) in addition to pathways involving ECM organization, ECM–ECM and ECM–cell interactions, and ECM remodeling. Interestingly, the lower levels in the hierarchy include a pathway involved in immune biology (interleukin-4 and interleukin-13 signaling).

**Table 1:** Select enriched reactome pathways identified by COL1A2 and/or LAMA3, listed in order of ascending *p*-values

Pathway name	*p* value	FDR*	Entities found
Anchoring fibril formation	1.96e-04	0.027	COL1A2, LAMA3
MET activates PTK2 signaling	8.80e-04	0.059	COL1A2, LAMA3
MET promotes cell motility	0.002	0.059	COL1A2, LAMA3
Non-integrin membrane-ECM interactions	0.003	0.084	COL1A2, LAMA3
Assembly of collagen fibrils and other multimeric structures	0.004	0.086	COL1A2, LAMA3
ECM proteoglycans	0.005	0.098	COL1A2, LAMA3
Collagen formation	0.009	0.111	COL1A2, LAMA3
Extracellular matrix organization	0.01	0.111	COL1A2, LAMA3, EMILIN1
Type I hemidesmosome assembly	0.015	0.111	LAMA3
GP1b-IX-V activation signaling	0.016	0.111	COL1A2
Degradation of the extracellular matrix	0.017	0.111	COL1A2, LAMA3
Platelet Adhesion to exposed collagen	0.022	0.111	COL1A2
Crosslinking of collagen fibrils	0.032	0.111	COL1A2
Interleukin-4 and Interleukin-13 signaling	0.033	0.111	COL1A2

^*^False discovery rate

### Histology

**Fig. 2:**
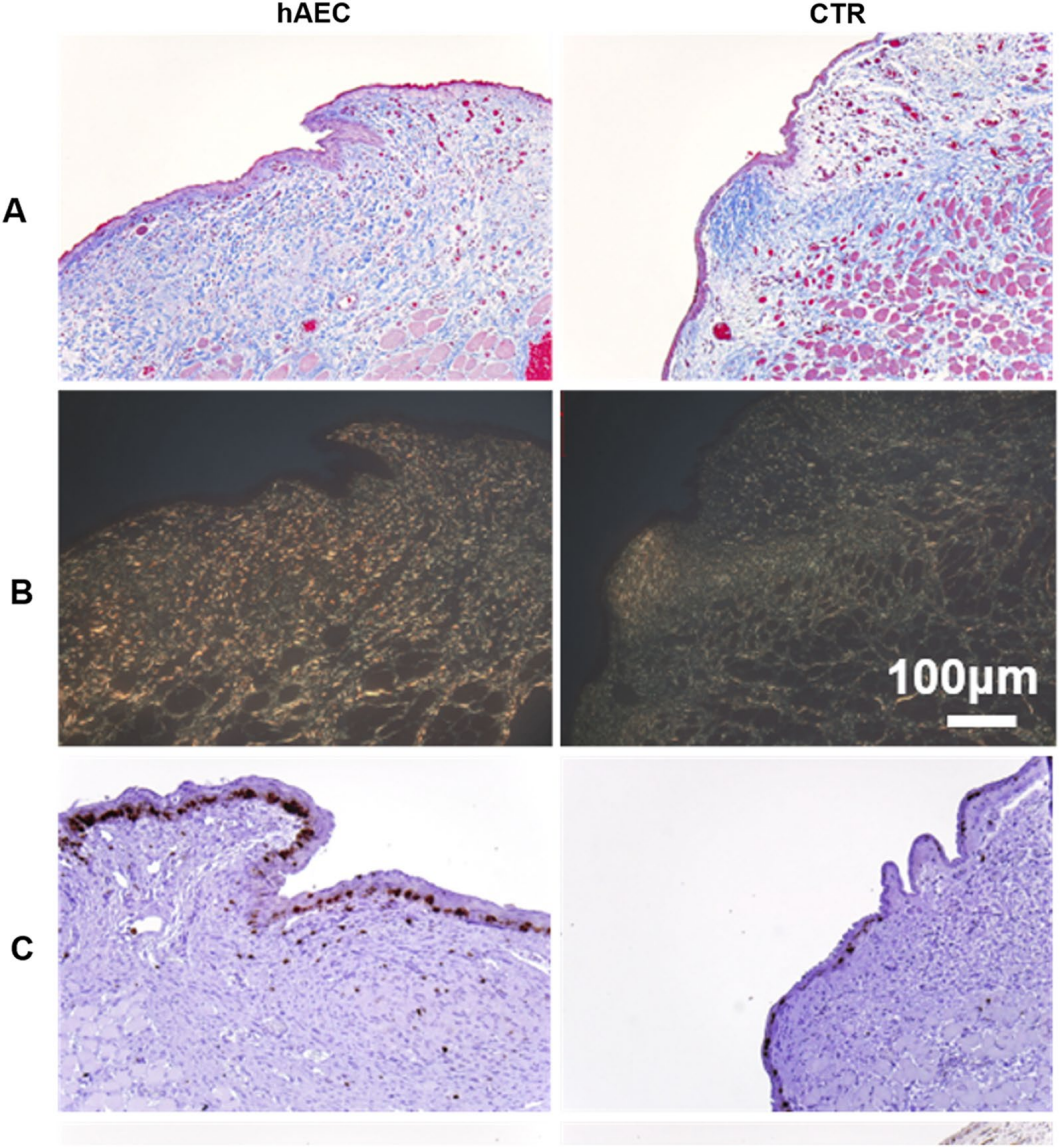
Histological analyses of treated (hAEC) and untreated (CTR) rabbit vocal folds. Collagen deposition is visualized by Masson trichrome (**A**) and picrosirius (polarized light) (**B**). Cell proliferation is assessed by immunohistochemical staining for Ki67 (**C**). Scale Bar = 100 μm for all panels

Trichrome staining showed connective tissue with more regular distribution and aligned connective tissue fibers in animals transplanted with hAEC, compared to the CTR groups (Fig. 2A). Histological analysis by polarized light on tissues stained with picrosirius red supports the better connective tissue fiber organization in the hAEC-treated group (Fig. 2B).

The quantification of the immunostaining for Ki67, a marker for proliferation (Fig. 2C), showed that the percents of Ki67+ cells in hAEC versus CTR groups were comparable in the stroma, but there was a trend (*p* = 0.07) toward a lower number of Ki67+ cells in the epithelium of hAEC-treated vocal folds vs. CTR (Table 2). This suggests a tendency toward a lower mitogenic activity of basal and parabasal cells in this group. Given the accepted negative relationship between the mitogenic activity of basal cells and the degree of differentiation process, these data supplement the finding of higher expression of keratin 1 in hAEC-treated vocal folds that is indicative of accelerated epithelial differentiation.

**Table 2:** Quantitative analyses of immunoreactivity of select markers in epithelium and/or lamina propria of the vocal fold

Measurement	Lamina propria (LP)			Epithelium		
	CTR	AEC	*p* value	CTR	AEC	*p* value
Ki67+ cells, %	65.2 (25.1)	63.1 (17.3)	0.9	238.0 (128.2)	76.6 (49.0)	**0.07**
αSMA+ cells, %	18.9 (20.7)	14.2 (4.9)	0.4			
Blood vessels, marked by CD31+ cells						
Density, % LP area	0.024 (0.003)	0.021 (0.008)	0.3			
Average lumen area, μm^2^	73 (21)	70 (45)	0.5			

Bold text denotes trend towards statistical significance

Values are mean (SD)

**Fig. 3:**
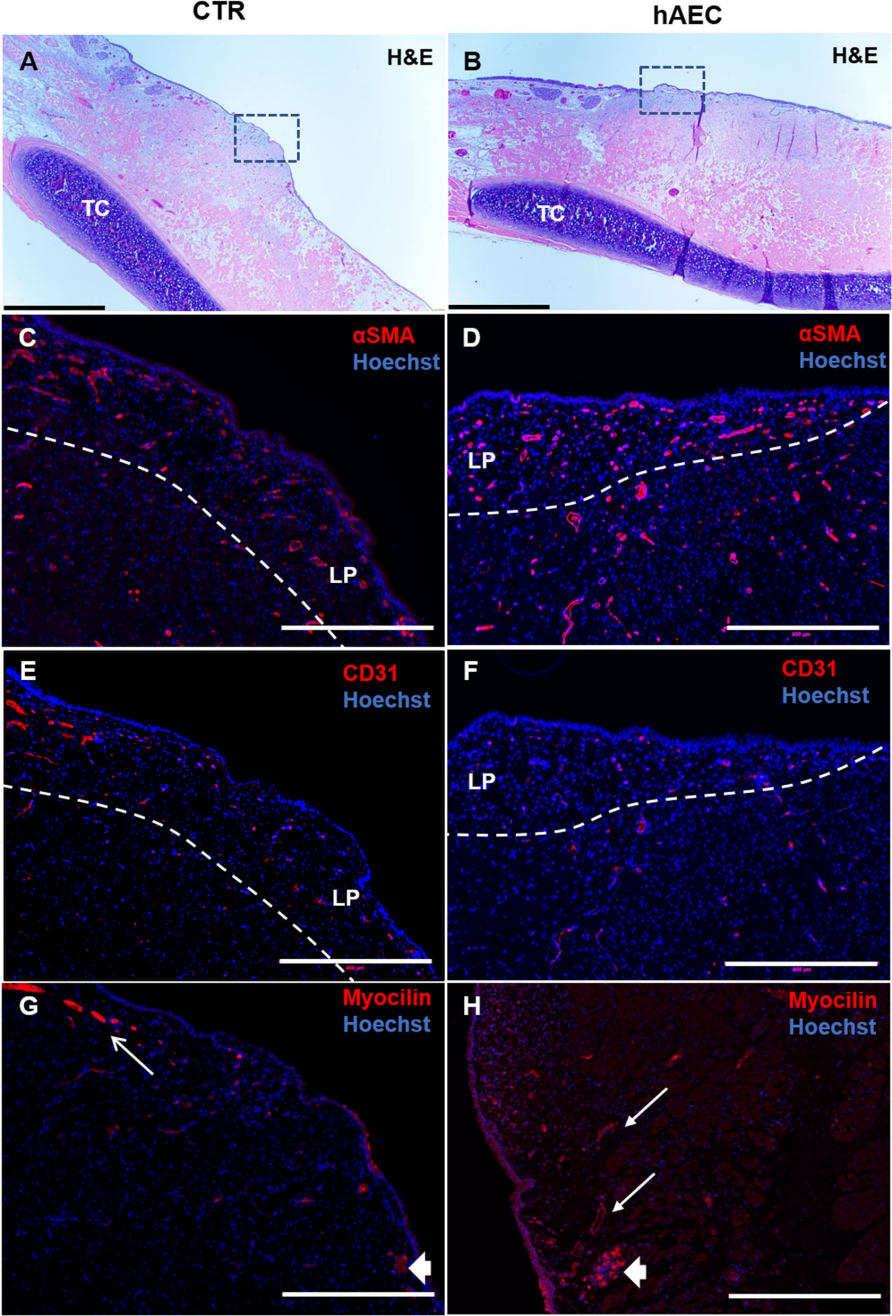
Immunofluorescence to evaluate granulation tissue and localization of myocilin by treatment group. The overall laryngeal topography including the vocal folds designated by the rectangles is shown in (**A**) and (**B**), TC, thyroid cartilage. Staining for α SMA (**C**, **D**) depicts vascular mural cells (pericytes and smooth muscle cells) and fibroblasts. Staining for CD31 (**E**, **F**) depicts endothelial cells of blood vessels; LP, lamina propria and the dashed line delineates its border with the underlying vocalis muscle. Myocilin is detected extracellularly around blood vessels (thin arrows) and in the adjacent cells of the mucous-serous glands (**G**, **H**) (thick arrow). Scale Bars = 1 mm (**A**, **B**) and 400 µm (**C**–**H**)

In contrast, the quantitative analyses of the αSMA- and CD31-stained images (Fig. 3C–F) did not find any difference in the fibroblast number and vascular features (Table 2), which was anticipated given the pilot nature of this study and the great degree of variability among the samples we noted.

In the rabbit vocal folds from both groups, a positive staining for myocilin (Fig. 3 G, H) was detected lightly along capillaries and around larger blood vessels (thin arrows) and more intensely within glandular secreting cells (thick arrow). The latter was further confirmed by staining of the adjacent glandular structures (Additional file 2: Fig. S1A–C), which were phenotypically characterized with PAS staining showing the presence of muco-substances intracellularly in magenta color (Additional file 2: Fig. S1B). In the human vocal fold (Additional file 2: Fig. S1D, E), the adventitia of arterioles and venules (thin arrows), as well as the basal membrane below the epithelium (thick arrows), were positively stained for myocilin, further supporting the extracellular localization of myocilin in the vocal folds.

## Discussion

To assess safety and therapeutic efficacy of hAEC, we analyzed the effects offered by hAEC in a classic experimental model for vocal fold damage, based on induced traumatic damage followed by local injection of xenogeneic stem cells in immunocompetent rabbits, with no immunosuppressants in support. We analyzed the protein profiles of the excised vocal folds using the methods of untargeted proteomics and pathway analysis to identify proteins whose abundance was altered by the hAEC treatment. Since this was an initial pilot study, we chose to perform these assays at one time point—at the beginning of the final ECM remodeling phase of healing (6 weeks post-injury), as it plays a critical role in restoring the pliability of the lamina propria.

This study identified several proteins that are upregulated and one protein that was downregulated by hAEC treatment. As the latter does not provide enough information to understand the potential biological consequences, we focus the discussion on the proteins with increased abundance. Among them are the core ECM proteins EMILIN1, LN, COL1 and a protein not previously reported in the vocal fold called myocilin. EMILIN1 is a homotrimeric glycoprotein from the EMILIN/multimerin family with diverse functions. It is produced by vascular mural cells and plays an important role in elastogenesis of the blood vessel wall providing an anchor for the endothelial and smooth muscle cells and promoting their proliferation [34]. The genetically or functional knockdown of EMILIN1 leads to reduced vascular cell proliferation and narrower blood vessels [35]. It is also produced by fibroblasts in dermal stroma [36] and notably by macrophages [37]*.* EMILIN1 interacts with components of the ECM, namely fibrillin and elastin, that together assemble into a complex network of elastic fibers which plays a pivotal role in the mechanical properties of the lamina propria and the quality of phonation [38, 39]. It also binds to components of the basement membrane, including collagen I and LN322, which are part of the anchoring complex [40]. EMILIN1 interacts with the TGFβ1 precursor complex and maintains TGFβ1 in a latent state [41] and, thereby, could suppress its mitogenic effects. In addition, EMILIN1 interacts directly with epithelial cells through α4β1 and α9β1 integrin receptors and inhibits their proliferation [36]. By these direct and indirect mechanisms, EMILIN1 could have played a role to lower the mitogenic activity of the basal cells we have observed. Taken together, we reason that the more abundant EMILIN1 may contribute to formation of stronger, and better organized elastic fiber mesh conferring superior elastic properties to the forming tissue and the blood vessels therein. By suppressing basal cell and fibroblast proliferation, EMILIN1 may accelerate epithelial cell differentiation and barrier development and temper the ECM protein production and produce a less fibrotic environment in the maturing tissue, respectively.

The primary statistical analysis showed that hAEC also increased (non-adjusted *p* < 0.05) the abundance of two other core proteins: (1) the α2 chain of the fibrous type I collagen (COL1), which together with two different and identical α1 chains form a triple helical structure, and (2) α3 chain of laminins, which is part of a few LNs including LN α3β3γ2 or LN 322. [Of note, LNs are heterotrimer glycoproteins composed of an α-, a β-, and a γ-chains; each type of chain has several subtypes: α (1–5), β (1–3), and γ (1–3).] We interpret that the increased expression of these subunits infers greater deposition of the respective complete proteins. Although the increased expression of both proteins lost significance after the more stringent statistical analyses, they both significantly stimulated the anchoring fibril formation pathway through distinct mechanisms. LN322 is secreted by epithelial and endothelial cells to act as the major adhesive component of the basement membrane, a sheet-like ECM that underlies them. LN322, in turn, anchors these cells through binding to the integrin receptors α3β1 and α6β4 that connect it to the keratin cytoskeleton [42]. During wound healing, these receptors are critical for regenerating an organized basement membrane that firmly anchor the cells. COL1 is mainly produced by myofibroblasts. However, alternatively activated (M2 phenotype) macrophages [43] are also a good source and the reactome analysis showed a tendency for enrichment of the IL-4 and IL13 pathway, known to induce the M2 phenotype of macrophages [44]. It is documented that COL1 is localized in the superficial (near the basement membrane) layer of lamina propria of normal human and rat vocal folds [45–47]. COL1 also participates in cell anchorage by binding to its specific integrin receptors, α1β1 and α2β1, and thereby strengthening cell bondage to the superficial layer of the lamina propria. Taken together, these findings suggest that hAEC may improve the tensile strength required around the basal membrane to maintain the vocal fold shape while withstanding vibratory forces.

In addition to the function of LN322 and COL-1 as mechanical linkers, through the integrins they also play an important role in signaling and regulation of the behavior of all cells found in this junctional area including epithelial cells, endothelial cells, and fibroblasts. For example, the collagen integrin receptor α1β1 promotes cell growth, whereas α2β1 mediates collagen gel contraction [48–50]. The finding of the tendency towards more active proliferation of basal cells, as judged by the higher Ki67 index, suggests active signaling through the α1β1 receptor. From the LN integrins, the α3β1 receptor appears to affect cell behavior [51]. The enrichment analysis shows that COL1 and laminin tend to (adjusted *p* = 0.06) activate signaling through a receptor tyrosine kinase c-met that binds to hepatocyte growth factor (HGF). It is documented that this is one of the mechanisms by which COL1 increases the angiogenic potential of the endothelial cells [52]. Notably, HGF has established antifibrotic efficacy in vocal fold wound healing in both in vitro and in vivo studies [53, 54].

A novel finding was that hAEC upregulate the expression of myocilin, a member of the family of olfactomedin domain-containing glycoproteins, as its presence in the vocal fold has not been reported. Myocilin is secreted into the aqueous humor of the eye by the trabecular meshwork cells and plays a role in regulating the intraocular pressure [55, 56]. It is also found in many non-ocular tissues, some of which do not secrete it, [57] suggesting that it has functions inside and outside of the cell. However, these functions are not exactly understood except that the structural olfactomedin domains facilitate protein–protein interactions, intercellular interactions, and cell adhesion [58]. Likewise, the factors regulating myocilin transcription are unknown. Interestingly, culturing trabecular meshwork on soft extracellular matrices enhances myocilin gene expression, [57] suggesting a potential role of hAEC in restoring softer tissue formation. An alternate proposition may be inferred from the premises that (1) myocilin can associate with the exosomal membranes from outside [59], (2) secreted exosomes are a part of the paracrine actions of hAEC [20], and (3) there is a high homology (84%) between human and rabbit myocilins. It is possible that hAEC-derived myocilin may account for the higher abundance of the myocilin in the rabbit hAEC-treated vocal folds.

We demonstrate that myocilin is localized outside of the blood vessels in both human and rabbit vocal folds and in the basal membrane of the human vocal fold. It is possible that myocilin may support vascular morphogenesis and provide more structural and organizational stability for the vascular endothelium, which is also reinforced by its capability to bind to structural components of the basement membrane and extracellular matrix including fibronectin, LN, and collagen I (reviewed in [60, 61]). We thus interpret the increased abundance of myocilin in the hAEC-treated group as evidence for regeneration of a more robust vascular network given that the number of blood vessels were comparable. The concomitant finding of myocilin positive staining of the glandular epithelium suggests a possible additional role in mucous secretion in the subglottic mucosa. Further work will be required to reveal the specific roles of myocilin in cell adhesion, wound healing and the biology of the vocal fold and the respiratory epithelium.

In addition to the changes in the expression of the ECM proteins, we found two upregulated proteins that illustrate an effect of hAEC on cellular function and fate. The first one is Rab GDP (guanosine diphosphate) dissociation inhibitor (GDI). Rab proteins are small guanine nucleotide-binding (G) proteins, which have an inherent enzymatic capability for GTP (guanosine triphosphate) hydrolysis (GTPases). Rab GDI regulates intracellular vesicular transport and their fusion with cellular membranes [62, 63] achieving the intracellular transport of newly synthesized proteins and sometimes their secretion. The role of exocytosis-mediated cell–matrix interactions and cell-to-cell communications has been shown to regulate reepithelization of decellularized skin biomatrix scaffolds [64] and all phases of cutaneous wound healing, including the collagen production and cross-linking during the ECM remodeling phase [60] and neovascularization [61, 65]. Taken together, our finding of increased expression of Rab GDI indicates that hAEC activate the vesicle endocytic and exocytic pathways to a greater degree. However, the processes and mechanisms by which it affects the vocal fold wound healing warrants further investigation.

The second upregulated protein is the hAEC increased expression of K1. K1 a member of the family of keratins, one of the six main groups of intermediate filaments in epithelial cells, that includes type I (acidic) keratins (numbered K9-K20) and type II (basic) keratins (numbered K1-K8). Epithelial cells frequently co-express one type I and one type II keratin heterodimer partners. Dynamic actin-myosin and keratin filament formations play a role during the migration of epithelial cells, which in rabbits is completed in approximately two weeks post-injury [66]. The setting at six weeks post-injury is, therefore, static where keratin establishes stable perinuclear cage-like structures linked to anchored keratin filaments in topologically restricted formations including tight junctions, adherens junctions, desmosomes connecting neighboring cells, and hemidesmosomes connecting the basal cells to the basement membrane [67–69]*.* This cytoskeletal reorganization provides epithelial stabilization. Additionally, basal cell polarization and apical cell emergence enable suprabasal cell formation and subsequent stratification [70]. The cells that migrate into the suprabasal layers enter a differentiation pathway when they downregulate the genes for K5/K14 pair, characteristic for the mitotically active basal cells, and upregulate the differentiation-specific genes including keratins K1 initially followed by its partner K10 [57, 58]. The more abundant K1 in the hAEC group suggests an accelerated induction of the differentiation program of the basal epithelial cells, start of stratification, and restoration of the barrier function. This is further supported by the tendency to a lower mitogenic activity of basal and parabasal cells in the hAEC-treated group as judged by the tendency towards lower percentage of the Ki67+ positive epithelial cells and the accepted inverse relationship between the rate of basal cell proliferation and epithelial cell differentiation. In skin, K1-positive suprabasal keratinocytes have also been shown to reduce inflammation by suppressing the production and release of the inflammatory cytokine IL-18 [71]*.* Therefore, the more abundant K1 in the hAEC-treated group may also contribute to a less inflammatory microenvironment that promotes more rapid healing with less scar formation.

This study is not without limitations. First, the proteomics data indicate that the injection of hAEC in injured vocal folds alters the ECM composition of the granulation tissue and basement membranes and epithelial cell behavior. However, our work did not determine the functional consequences of these changes including the integrity of the physical barrier of epithelium and the mechanical and the aerodynamic properties of the lamina propria. Second, we performed a pathway analysis of the significant proteomics data to address mechanisms of interactions among them and an established pathway of the anchoring fibril formation emerged. However, this work did not test the validity of this pathway’s involvement in migration, proliferation, and differentiation of fibroblasts and epithelial or endothelial cells. Finally, we noticed that soluble mediators such as growth factors, cytokines, and enzymes were not detected in our study indicating a possible loss of proteins that are loosely bound to other ECM proteins through polyionic bonds during the protein extraction.

## Conclusion

This pilot study found that injection of hAEC into an injured rabbit vocal fold increased the abundance of ECM proteins that play an important role in elastic fiber formation, regulation of tensile strength, and basement membrane cell anchorage, as well as proteins that reflect increased cellular activity and epithelial cell differentiation. These data suggest a beneficial effect of hAEC on the reciprocal ECM–cell interactions, where altered cell secretion of ECM and basement membrane proteins create a microenvironment that accelerates differentiation of regenerated epithelium and promotes neovascularization indicative of accelerated repair. Further studies will use gain or loss of function approaches to investigate the validity of individual ECM components and cell anchorage pathways in regulating the behavior of epithelial cells, endothelial cells, and fibroblasts. Future studies should also evaluate hAEC optimization of the barrier function of regenerated epithelium and the mechanical properties of forming tissue at both early (6 weeks) and late (6 months) time points [67]. Lastly, additional steps or methods could be considered for a more integrated ECM map, such as an extraction of loosely bound proteins using high salt solution [68], complementary cytokine array assays, or higher-resolution mass spectrometry.

## Supplementary Information

**Additional file 1.** Pathway analysis report.

**Additional file 2: Fig. S1.** Immunofluorescence for myocilin in submucosal gland adjacent to the vocal fold in a rabbit larynx and in human vocal fold. Representative images of the glandular epithelium adjacent to the vocal fold show its general histology with H&E staining (**A**), the presence of muco-substances intracellularly with PAS staining (**B** magenta color) and the expression of myocilin in the glandular secreting cells with immunofluorescence (**C**). In the human vocal fold (**D**, **E**), the myocilin-positive sites are the adventitia of arterioles (A) and venules (V) (thin arrows) and the basal membrane (thick arrows). Scale Bars: 200 µm (**A**–**C**), 100 µm (**D** insert), and 20 µm (**E**).

## Data Availability

All data generated or analyzed during this study are included in this published article [and its supplementary information files]. The raw data from Mass Spectrometry are available in the MassIVE repository: ftp://massive.ucsd.edu/MSV000087132/.

## Abbreviations

hAEC: Human amnionic epithelial cells
ECM: Extracellular matrix
CTR: Control
HGF: Hepatocyte growth factor
COL1: Collagen 1
LN: Laminin
MECs: Myoepithelial cells
