# Supplementary material for: Human amniotic epithelial cell transplantation improves scar remodeling in a rabbit model of acute vocal fold injury: a pilot study
Source: Stem Cell Res Ther. 2022 Jan 25;13:31. doi: 10.1186/s13287-022-02701-w (PMC8787902; doi:10.1186/s13287-022-02701-w)
Supplement: Supplementary file 1 — Additional file 1. Pathway analysis report. [file 13287_2022_2701_MOESM1_ESM.pdf]

reactome

Pathway Analysis Report

This report contains the pathway analysis results for the submitted sample ''. Analysis was performed against Reactome version 73 on 28/09/2020. The web link to these results is:

https://reactome.org/PathwayBrowser/#/ANALYSIS=MjAyMDA5MjgwMzAxMDNfMTU0MA%3D%3D

Please keep in mind that analysis results are temporarily stored on our server. The storage period depends on usage of the service but is at least 7 days. As a result, please note that this URL is only valid for a limited time period and it might have expired.

Table of Contents

1. Introduction
2. Properties
3. Genome-wide overview
4. Most significant pathways
5. Pathways details
6. Identifiers found
7. Identifiers not found

1. Introduction

Reactome is a curated database of pathways and reactions in human biology. Reactions can be considered as pathway 'steps'. Reactome defines a 'reaction' as any event in biology that changes the state of a biological molecule. Binding, activation, translocation, degradation and classical biochemical events involving a catalyst are all reactions. Information in the database is authored by expert biologists, entered and maintained by Reactome’s team of curators and editorial staff. Reactome content frequently cross-references other resources e.g. NCBI, Ensembl, UniProt, KEGG (Gene and Compound), ChEBI, PubMed and GO. Orthologous reactions inferred from annotation for Homo sapiens are available for 17 non-human species including mouse, rat, chicken, puffer fish, worm, fly, yeast, rice, and Arabidopsis. Pathways are represented by simple diagrams following an SBGN-like format.

Reactome's annotated data describe reactions possible if all annotated proteins and small molecules were present and active simultaneously in a cell. By overlaying an experimental dataset on these annotations, a user can perform a pathway over-representation analysis. By overlaying quantitative expression data or time series, a user can visualize the extent of change in affected pathways and its progression. A binomial test is used to calculate the probability shown for each result, and the p-values are corrected for the multiple testing (Benjamini–Hochberg procedure) that arises from evaluating the submitted list of identifiers against every pathway.

To learn more about our Pathway Analysis, please have a look at our relevant publications:

Fabregat A, Sidiropoulos K, Garapati P, Gillespie M, Hausmann K, Haw R, … D’Eustachio P (2016). The reactome pathway knowledgebase. Nucleic Acids Research, 44(D1), D481–D487. https://doi.org/10.1093/nar/gkv1351.

Fabregat A, Sidiropoulos K, Viteri G, Forner O, Marin-Garcia P, Arnau V, … Hermjakob H (2017). Reactome pathway analysis: a high-performance in-memory approach. BMC Bioinformatics, 18.

2. Properties

- This is an **overrepresentation** analysis: A statistical (hypergeometric distribution) test that determines whether certain Reactome pathways are over-represented (enriched) in the submitted data. It answers the question 'Does my list contain more proteins for pathway X than would be expected by chance?' This test produces a probability score, which is corrected for false discovery rate using the Benjamani-Hochberg method.
- 13 out of 16 identifiers in the sample were found in Reactome, where 135 pathways were hit by at least one of them.
- All non-human identifiers have been converted to their human equivalent.
- This report is filtered to show only results for species 'Homo sapiens' and resource 'all resources'.
- The unique ID for this analysis (token) is MjAyMDA5MjgwMzAxMDNfMTU0MA%3D%3D. This ID is valid for at least 7 days in Reactome's server. Use it to access Reactome services with your data.

3. Genome-wide overview

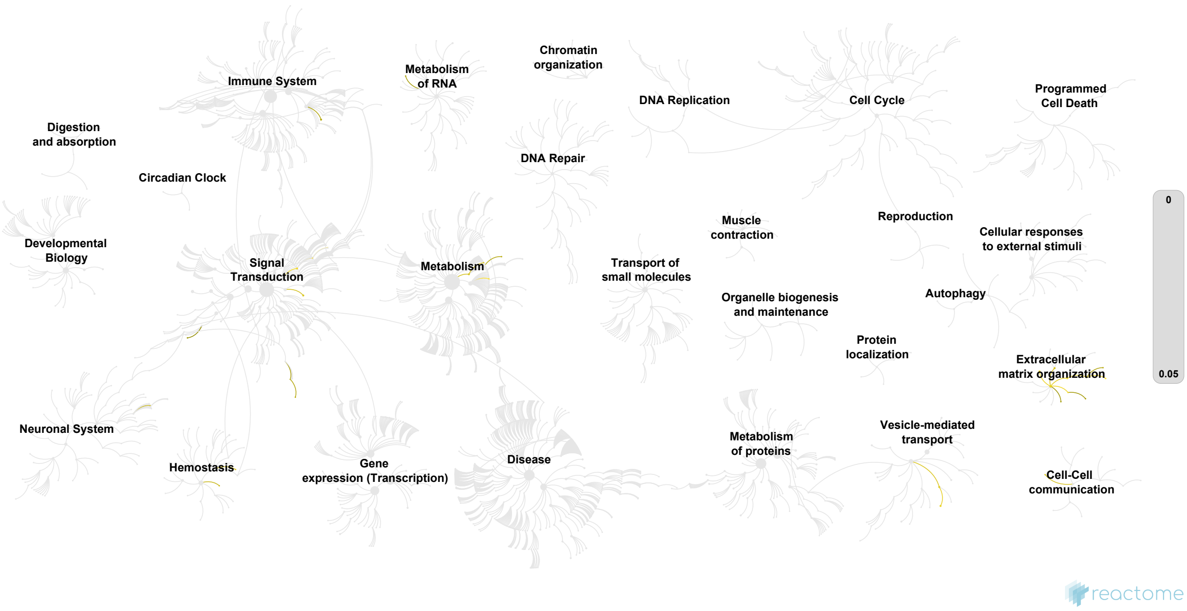

This figure shows a genome-wide overview of the results of your pathway analysis. Reactome pathways are arranged in a hierarchy. The center of each of the circular "bursts" is the root of one top-level pathway, for example "DNA Repair". Each step away from the center represents the next level lower in the pathway hierarchy. The color code denotes over-representation of that pathway in your input dataset. Light grey signifies pathways which are not significantly over-represented.

4. Most significant pathways

The following table shows the 25 most relevant pathways sorted by p-value.

Pathway name	Entities				Reactions	
	found	ratio	p-value	FDR*	found	ratio
Anchoring fibril formation	2 / 15	0.001	1.96e-04	0.027	1 / 4	3.09e-04
MET activates PTK2 signaling	2 / 32	0.002	8.80e-04	0.059	1 / 5	3.86e-04
Glutamate and glutamine metabolism	2 / 40	0.003	0.001	0.059	1 / 14	0.001
MET promotes cell motility	2 / 45	0.003	0.002	0.059	1 / 12	9.26e-04
Non-integrin membrane-ECM interactions	2 / 61	0.004	0.003	0.084	5 / 22	0.002
Assembly of collagen fibrils and other multimeric structures	2 / 67	0.005	0.004	0.086	16 / 26	0.002
ECM proteoglycans	2 / 79	0.005	0.005	0.098	7 / 23	0.002
Signaling by MET	2 / 88	0.006	0.006	0.108	1 / 49	0.004
RAB GEFs exchange GTP for GDP on RABs	2 / 94	0.006	0.007	0.108	18 / 21	0.002
Collagen formation	2 / 104	0.007	0.009	0.111	37 / 77	0.006
Extracellular matrix organization	3 / 329	0.022	0.01	0.111	70 / 318	0.025
Rab regulation of trafficking	2 / 129	0.009	0.013	0.111	18 / 35	0.003
Type I hemidesmosome assembly	1 / 11	7.50e-04	0.015	0.111	4 / 6	4.63e-04
AMPK inhibits chREBP transcriptional activation activity	1 / 11	7.50e-04	0.015	0.111	2 / 4	3.09e-04
GP1b-IX-V activation signalling	1 / 12	8.18e-04	0.016	0.111	5 / 7	5.40e-04
Rho GTPase cycle	2 / 144	0.01	0.016	0.111	2 / 5	3.86e-04
Integration of energy metabolism	2 / 145	0.01	0.016	0.111	4 / 62	0.005
Degradation of the extracellular matrix	2 / 148	0.01	0.017	0.111	7 / 105	0.008
Platelet Adhesion to exposed collagen	1 / 16	0.001	0.022	0.111	4 / 6	4.63e-04
CREB1 phosphorylation through the activation of Adenylate Cyclase	1 / 17	0.001	0.023	0.111	2 / 6	4.63e-04
PKA activation	1 / 23	0.002	0.031	0.111	2 / 4	3.09e-04
PKA activation in glucagon signalling	1 / 23	0.002	0.031	0.111	1 / 2	1.54e-04
Crosslinking of collagen fibrils	1 / 24	0.002	0.032	0.111	11 / 13	0.001
Interleukin-4 and Interleukin-13 signaling	2 / 211	0.014	0.033	0.111	1 / 46	0.004
PKA-mediated phosphorylation of CREB	1 / 26	0.002	0.035	0.111	2 / 7	5.40e-04

* False Discovery Rate

5. Pathways details

For every pathway of the most significant pathways, we present its diagram, as well as a short summary, its bibliography and the list of inputs found in it.

1. Anchoring fibril formation (R-HSA-2214320)

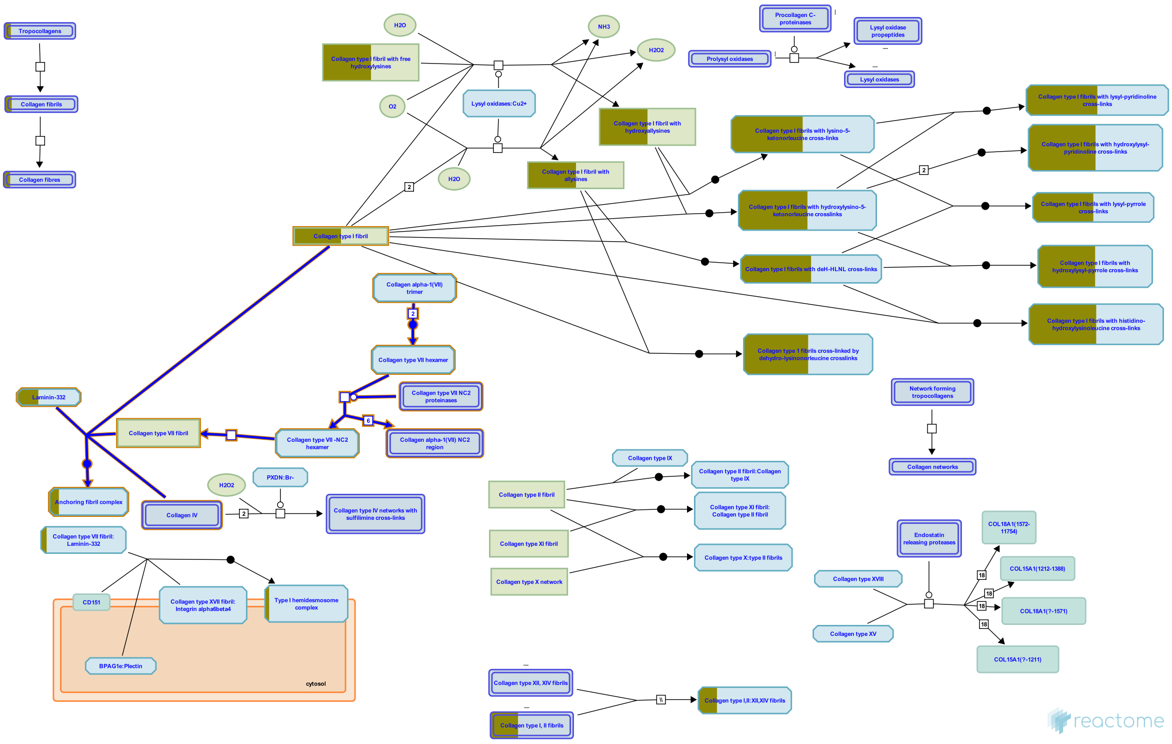

Cellular compartments: extracellular region.

Collagen VII forms anchoring fibrils, composed of antiparallel dimers that connect the dermis to the epidermis (Bruckner-Tuderman 2009, Has & Kern 2010). During fibrillogenesis, the nascent type VII procollagen molecules dimerize in an antiparallel manner. The C-propeptide is then removed by Bone morphogenetic protein 1 (Rattenholl et al. 2002) and the processed antiparallel dimers laterally aggregate (Villone et al. 2008, Gordon & Hahn 2010).

References

Chung HJ & Uitto J (2010). Type VII collagen: the anchoring fibril protein at fault in dystrophic epidermolysis bullosa. Dermatol Clin, 28, 93-105.

Edit history

Date	Action	Author
2012-04-30	Authored	Jupe S
2012-04-30	Created	Jupe S
2012-10-08	Reviewed	Kalamajski S, Raleigh S
2012-11-12	Edited	Jupe S
2012-11-19	Reviewed	Ricard-Blum S

Date	Action	Author
2020-05-28	Modified	Cook J

Entities found in this pathway (2)

Input	UniProt Id	Input	UniProt Id
COL1A2	P08123	LAMA3	Q16787

2. MET activates PTK2 signaling (R-HSA-8874081)

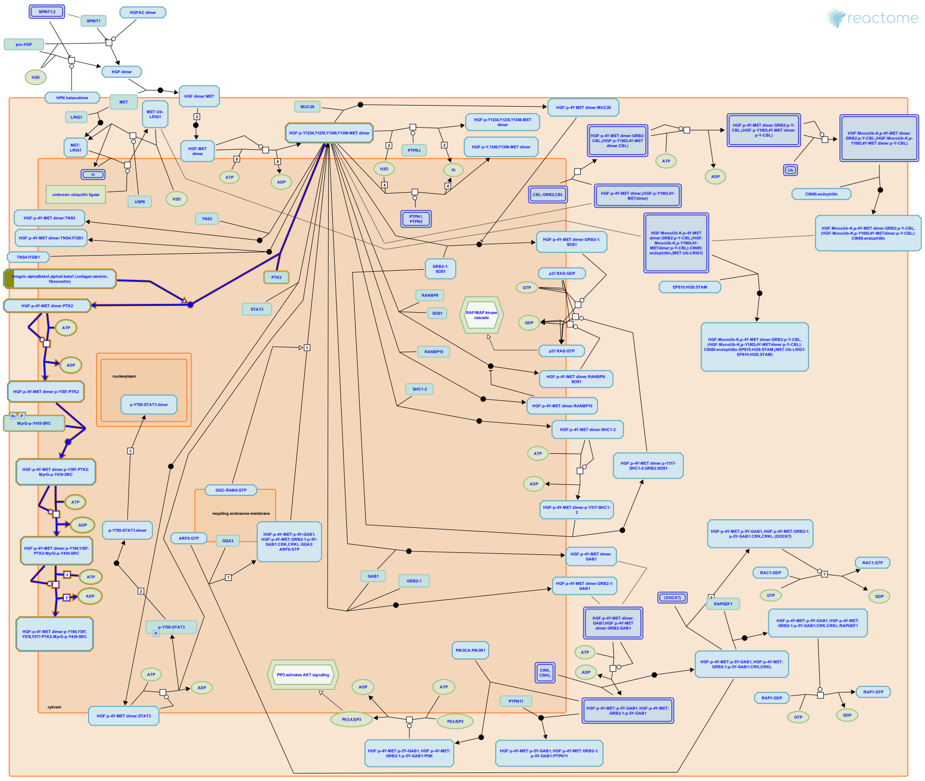

MET receptor activates the focal adhesion kinase PTK2 (FAK1) in a process that depends on the simultaneous interaction of PTK2 with integrins and with MET. SRC is needed for PTK2 to become fully active. Activation of PTK2 is needed for HGF-induced cell motility (Beviglia et al. 1999, Parr et al. 2001, Chen and Chen 2006, Lietha et al. 2007, Chen et al. 2011, Brami-Cherrier et al. 2014).

References

Beviglia L & Kramer RH (1999). HGF induces FAK activation and integrin-mediated adhesion in MTLn3 breast carcinoma cells. Int. J. Cancer, 83, 640-9.

Chen SY & Chen HC (2006). Direct interaction of focal adhesion kinase (FAK) with Met is required for FAK to promote hepatocyte growth factor-induced cell invasion. Mol. Cell. Biol., 26, 5155-67.

Parr C, Davies G, Nakamura T, Matsumoto K, Mason MD & Jiang WG (2001). The HGF/SF-induced phosphorylation of paxillin, matrix adhesion, and invasion of prostate cancer cells were suppressed by NK4, an HGF/SF variant. Biochem. Biophys. Res. Commun., 285, 1330-7.

Brami-Cherrier K, Gervasi N, Arsenieva D, Walkiewicz K, Boutterin MC, Ortega A, ... Arold ST (2014). FAK dimerization controls its kinase-dependent functions at focal adhesions. EMBO J., 33, 356-70.

Lietha D, Cai X, Ceccarelli DF, Li Y, Schaller MD & Eck MJ (2007). Structural basis for the autoinhibition of focal adhesion kinase. Cell, 129, 1177-87.

Edit history

Date	Action	Author
2016-05-20	Created	Orlic-Milacic M

Date	Action	Author
2016-06-14	Edited	Orlic-Milacic M
2016-06-14	Authored	Orlic-Milacic M
2016-07-11	Reviewed	Heynen G, Birchmeier W
2020-06-04	Modified	Cook J

Entities found in this pathway (2)

Input	UniProt Id	Input	UniProt Id
COL1A2	P08123	LAMA3	Q16787

3. Glutamate and glutamine metabolism (R-HSA-8964539)

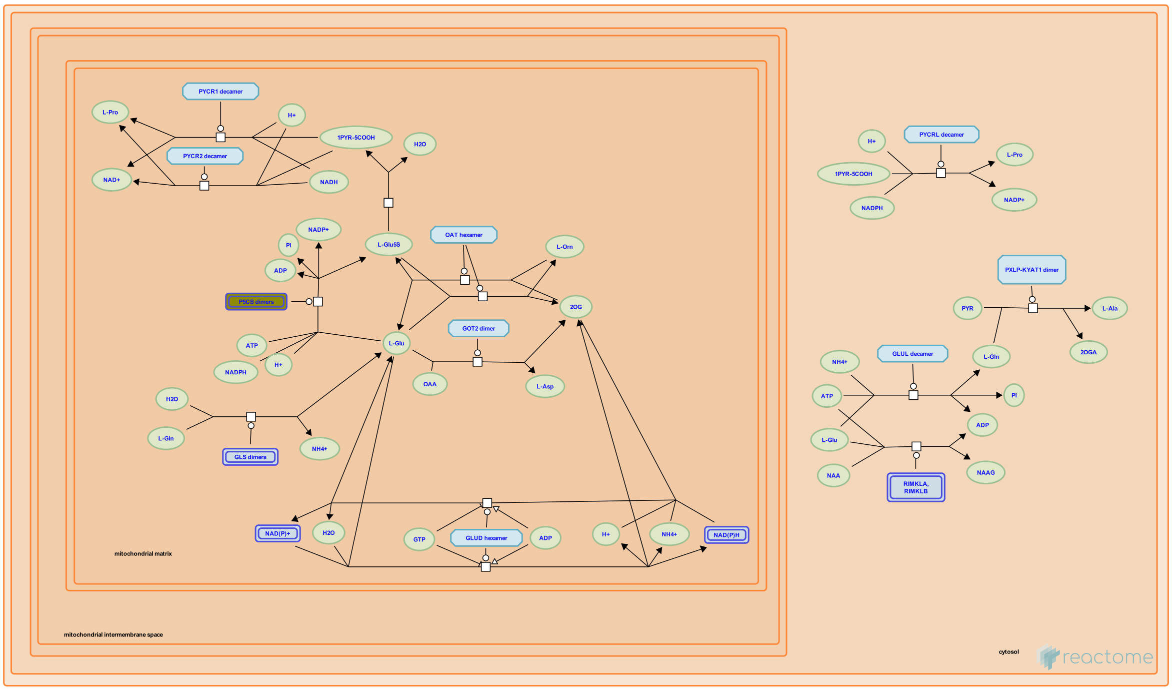

These reactions mediate the synthesis of glutamate and glutamine from ammonia and TCA cycle intermediates and allow the utilization of the carbon atoms from these amino acids for glucose synthesis under fasting conditions. These reactions also provide a means to collect nitrogen, both as ammonia and as amino groups, and direct it towards urea synthesis. Transamination, the conversion of an amino acid to the corresponding alpha-keto acid coupled to the conversion of a molecule of 2-oxoglutarate (alpha-ketoglutarate) to glutamate, is the first step in the catabolism of most amino acids. Transamination reactions are freely reversible so they also provide a means to balance concentrations of various amino acids and 2-oxo (alpha-keto) acids in the cell (Felig 1975; Häussinger 1990; Owen et al. 1979).

References

Felig P (1975). Amino acid metabolism in man. Annu. Rev. Biochem., 44, 933-55.

Häussinger D (1990). Liver glutamine metabolism. JPEN J Parenter Enteral Nutr, 14, 56S-62S.

Owen OE, Reichard GA, Patel MS & Boden G (1979). Energy metabolism in feasting and fasting. Adv. Exp. Med. Biol., 111, 169-88.

Edit history

Date	Action	Author
2017-02-10	Authored	Jassal B
2017-02-17	Created	Jassal B
2017-03-22	Edited	D'Eustachio P
2020-06-04	Modified	Cook J

Entities found in this pathway (1)

Input	UniProt Id
ALDH18A1	P54886-1, P54886-2

4. MET promotes cell motility (R-HSA-8875878)

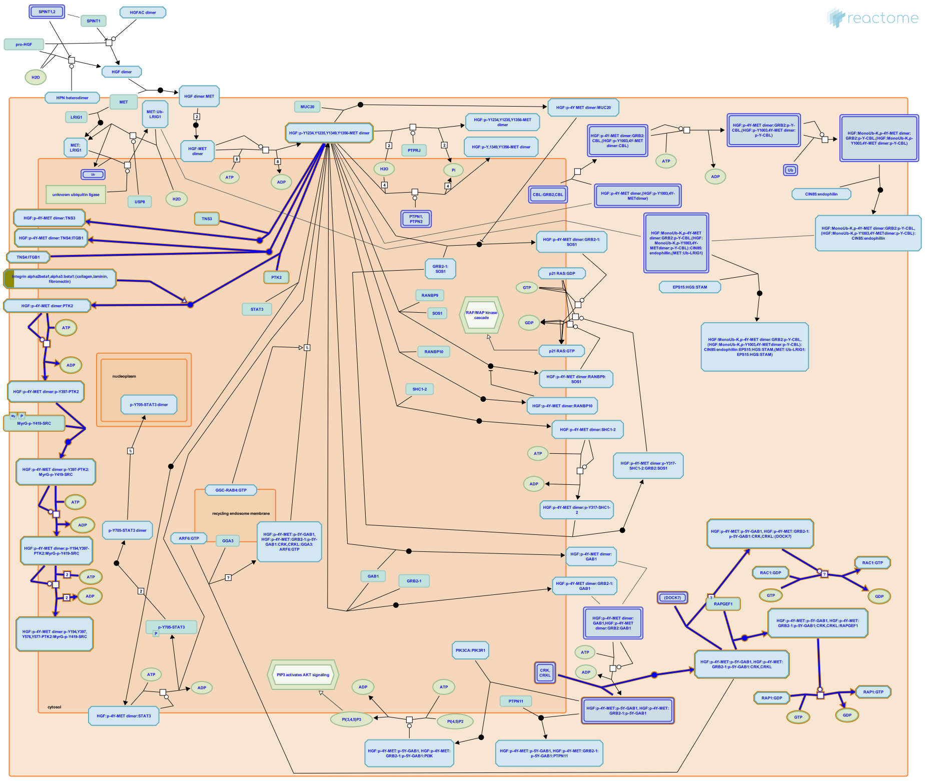

Direct and indirect interactions of MET with integrins, focal adhesion kinase PTK2 (FAK1), tensin-4 (TNS4) and GTPases RAP1 and RAC1, induce morphological changes that promote cell motility and play an important role in HGF-induced invasiveness of cancer cells (Weidner et al. 1993, Beviglia et al. 1999, Sakkab et al. 2000, Parr et al. 2001, Trusolino et al. 2001, Lamorte et al. 2002, Chen and Chen 2006, Watanabe et al. 2006, Muharram et al. 2014, Murray et al. 2014).

References

Beviglia L & Kramer RH (1999). HGF induces FAK activation and integrin-mediated adhesion in MTLn3 breast carcinoma cells. Int. J. Cancer, 83, 640-9.

Sakkab D, Lewitzky M, Posern G, Schaeper U, Sachs M, Birchmeier W & Feller SM (2000). Signaling of hepatocyte growth factor/scatter factor (HGF) to the small GTPase Rap1 via the large docking protein Gab1 and the adapter protein CRKL. J. Biol. Chem., 275, 10772-8.

Parr C, Davies G, Nakamura T, Matsumoto K, Mason MD & Jiang WG (2001). The HGF/SF-induced phosphorylation of paxillin, matrix adhesion, and invasion of prostate cancer cells were suppressed by NK4, an HGF/SF variant. Biochem. Biophys. Res. Commun., 285, 1330-7.

Lamorte L, Royal I, Naujokas M & Park M (2002). Crk adapter proteins promote an epithelial-mesenchymal-like transition and are required for HGF-mediated cell spreading and breakdown of epithelial adherens junctions. Mol. Biol. Cell, 13, 1449-61.

Chen SY & Chen HC (2006). Direct interaction of focal adhesion kinase (FAK) with Met is required for FAK to promote hepatocyte growth factor-induced cell invasion. Mol. Cell. Biol., 26, 5155-67.

Edit history

Date	Action	Author
2016-06-09	Created	Orlic-Milacic M
2016-06-14	Edited	Orlic-Milacic M
2016-06-14	Authored	Orlic-Milacic M
2016-07-11	Reviewed	Heynen G, Birchmeier W
2020-05-29	Modified	Cook J

Entities found in this pathway (2)

Input	UniProt Id	Input	UniProt Id
COL1A2	P08123	LAMA3	Q16787

5. Non-integrin membrane-ECM interactions (R-HSA-3000171)

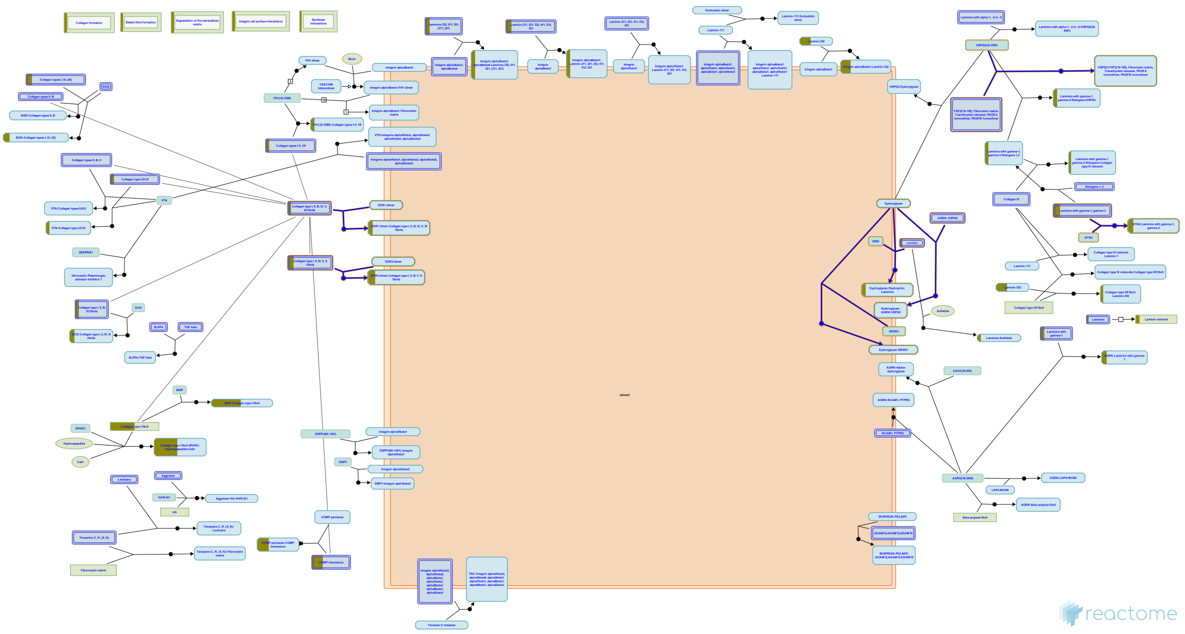

Several non-integrin membrane proteins interact with extracellular matrix proteins. Transmembrane proteoglycans may associate with integrins and growth factor receptors to influence their function, or they can signal independently, often influencing the actin cytoskeleton.

References

Rosso F, Giordano A, Barbarisi M & Barbarisi A (2004). From cell-ECM interactions to tissue engineering. J. Cell. Physiol., 199, 174-80.

Couchman JR (2010). Transmembrane signaling proteoglycans. Annu. Rev. Cell Dev. Biol., 26, 89-114.

Edit history

Date	Action	Author
2012-07-31	Authored	Jupe S
2013-01-24	Created	Jupe S
2013-04-26	Edited	Jupe S
2013-05-22	Reviewed	Ricard-Blum S
2020-05-29	Modified	Cook J

Entities found in this pathway (2)

Input	UniProt Id	Input	UniProt Id
COL1A2	P08123	LAMA3	Q16787

6. Assembly of collagen fibrils and other multimeric structures (R-HSA-2022090)

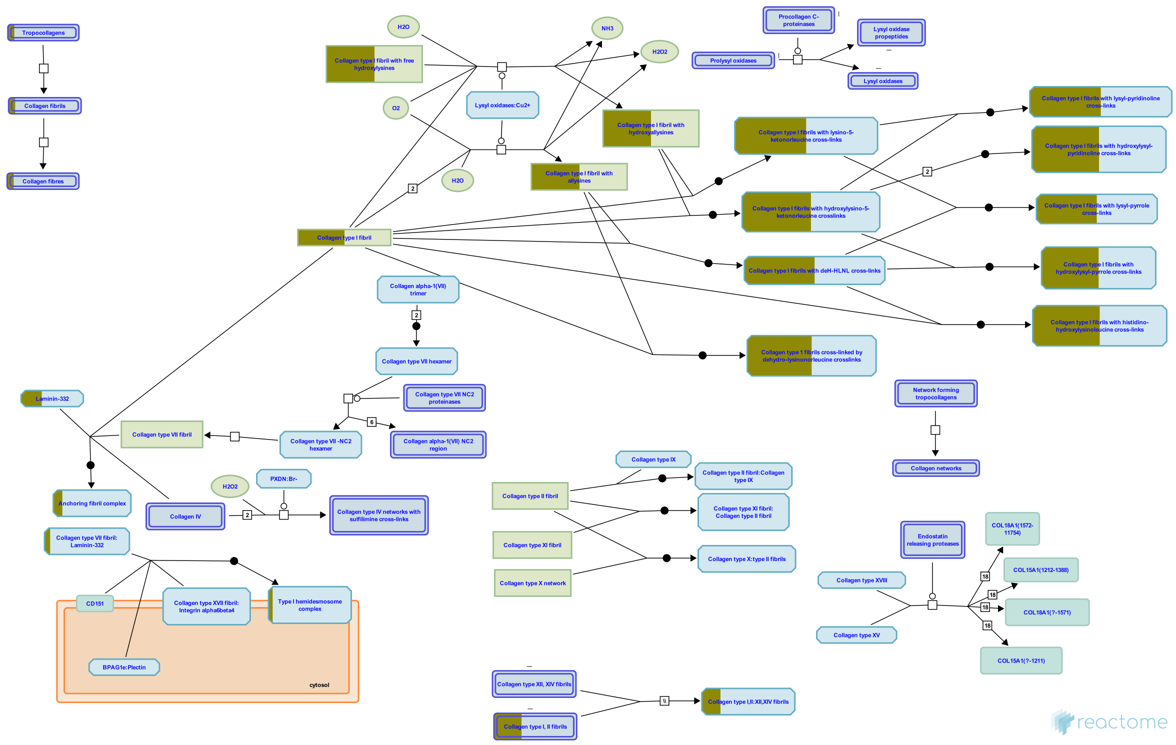

Collagen trimers in triple-helical form, referred to as procollagen or collagen molecules, are exported from the ER and trafficked through the Golgi network before secretion into the extracellular space. For fibrillar collagens namely types I, II, III, V, XI, XXIV and XXVII (Gordon & Hahn 2010, Ricard-Blum 2011) secretion is concomitant with processing of the N and C terminal collagen propeptides. These processed molecules are known as tropocollagens, considered to be the units of higher order collagen structures. They form within the extracellular space via a process that can proceed spontaneously, but in the cellular environment is regulated by many collagen binding proteins such as the FACIT (Fibril Associated Collagens with Interrupted Triple helices) family collagens and Small Leucine-Rich Proteoglycans (SLRPs). The architecture formed ultimately depends on the collagen subtype and the cellular conditions. Structures include the well-known fibrils and fibres formed by the major structural collagens type I and II plus several different types of supramolecular assembly (Bruckner 2010). The mechanical and physical properties of tissues depend on the spatial arrangement and composition of these collagen-containing structures (Kadler et al. 1996, Shoulders & Raines 2009, Birk & Bruckner 2011).

Fibrillar collagen structures are frequently heterotypic, composed of a major collagen type in association with smaller amounts of other types, e.g. type I collagen fibrils are associated with types III and V, while type II fibrils frequently contain types IX and XI (Wess 2005). Fibres composed exclusively of a single collagen type probably do not exist, as type I and II fibrils require collagens V and XI respectively as nucleators (Kadler et al. 2008, Wenstrup et al. 2011). Much of the structural understanding of collagen fibrils has been obtained with fibril-forming collagens, particularly type I, but some central features are believed to apply to at least the other fibrillar collagen subtypes (Wess 2005). Fibril diameter and length varies considerably, depending on the tissue and collagen types (Fang et al. 2012). The reasons for this are poorly understood (Wess 2005).

Some tissues such as skin have fibres that are approximately the same diameter while others such as tendon or cartilage have a bimodal distribution of thick and thin fibrils. Mature type I collagen fibrils in tendon are up to 1 cm in length, with a diameter of approx. 500 nm. An individual fibrillar collagen triple helix is less than 1.5 nm in diameter and around 300 nm long; collagen molecules must assemble to give rise to the higher-order fibril structure, a process known as fibrillogenesis, prevented by the presence of C-terminal propeptides (Kadler et al. 1987). In electron micrographs, fibrils have a banded appearance, due to regular gaps where fewer collagen molecules overlap, which occur because the fibrils are aligned in a quarter-stagger arrangement (Hodge & Petruska 1963). Collagen microfibrils are believed to have a quasi-hexagonal unit cell, with tropocollagen arranged to form supertwisted, right-handed microfibrils that interdigitate with neighbouring microfibrils, leading to a spiral-like structure for the mature collagen fibril (Orgel et al. 2006, Holmes & Kadler 2006).

Neighbouring tropocollagen monomers interact with each other and are cross-linked covalently by lysyl oxidase (Orgel et al. 2000, Maki 2006). Mature collagen fibrils are stabilized by lysyl oxidase-mediated cross-links. Hydroxylysyl pyridinoline and lysyl pyridinoline cross-links form between (hydroxy) lysine and hydroxylysine residues in bone and cartilage (Eyre et al. 1984). Arginoline cross-links can form in cartilage (Eyre et al. 2010); mature bovine articular cartilage contains roughly equimolar amounts of arginoline and hydroxylysyl pyridinoline based on peptide yields. Mature collagen fibrils in skin are stabilized by the lysyl oxidase-mediated cross-link histidino-hydroxylysinonorleucine (Yamauch et al. 1987). Due to the quarter-staggered arrangement of collagen molecules in a fibril, telopeptides most often interact with the triple helix of a neighbouring collagen molecule in the fibril, except for collagen molecules in register staggered by 4D from another collagen molecule. Fibril aggregation in vitro can be unipolar or bipolar, influenced by temperature and levels of C-proteinase, suggesting a role for the N- and C- propeptides in regulation of the aggregation process (Kadler et al. 1996). In vivo, collagen molecules at the fibril surface may retain their N-propeptides, suggesting that this may limit further accretion, or alternatively represents a transient stage in a model whereby fibrils grow in diameter through a cycle of deposition, cleavage and further deposition (Chapman 1989).

In vivo, fibrils are often composed from more than one type of collagen. Type III collagen is found associated with type I collagen in dermal fibrils, with the collagen III on the periphery, suggesting a regulatory role (Fleischmajer et al. 1990). Type V collagen associates with type I collagen fibrils, where it may limit fibril diameter (Birk et al. 1990, White et al. 1997). Type IX associates with the surface of narrow diameter collagen II fibrils in cartilage and the cornea (Wu et al. 1992, Eyre et al. 2004). Highly specific patterns of crosslinking sites suggest that collagen IX functions in interfibrillar networking (Wess 2005). Type XII and XIV collagens are localized near the surface of banded collagen I fibrils (Nishiyama et al. 1994). Certain fibril-associated collagens with interrupted triple helices (FACITs) associate with the surface of collagen fibrils, where they may serve to limit fibril fusion and thereby regulate fibril diameter (Gordon & Hahn 2010). Collagen XV, a member of the multiplexin family, is almost exclusively associated with the fibrillar collagen network, in very close proximity to the basement membrane. In human tissues collagen XV is seen linking banded collagen fibers subjacent to the basement membrane (Amenta et al. 2005). Type XIV collagen, SLRPs and discoidin domain receptors also regulate fibrillogenesis (Ansorge et al. 2009, Kalamajski et al. 2010, Flynn et al. 2010).

Collagen IX is cross-linked to the surface of collagen type II fibrils (Eyre et al. 1987). Type XII and XIV collagens are found in association with type I (Walchli et al. 1994) and type II (Watt et al. 1992, Eyre 2002) fibrils in cartilage. They are thought to associate non-covalently via their COL1/NC1 domains (Watt et al. 1992, Eyre 2002).

Some non-fibrillar collagens form supramolecular assemblies that are distinct from typical fibrils. Collagen VII forms anchoring fibrils, composed of antiparallel dimers that connect the dermis to the epidermis (Bruckner-Tuderman 2009). During fibrillogenesis, the nascent type VII procollagen molecules dimerize in an antiparallel manner. The C-propeptides are then removed by Bone morphogenetic protein 1 (Rattenholl et al. 2002) and the processed antiparallel dimers aggregate laterally. Collagens VIII and X form hexagonal networks and collagen VI forms beaded filament (Gordon & Hahn 2010, Ricard-Blum et al. 2011).

References

Kadler KE, Holmes DF, Trotter JA & Chapman JA (1996). Collagen fibril formation. Biochem J, 316, 1-11.

Orgel JP, San Antonio JD & Antipova O (2011). Molecular and structural mapping of collagen fibril interactions. Connect. Tissue Res., 52, 2-17.

Edit history

Date	Action	Author
2011-08-05	Authored	Jupe S
2011-11-25	Created	Jupe S
2012-10-08	Reviewed	Kalamajski S, Raleigh S
2012-11-12	Edited	Jupe S
2012-11-19	Reviewed	Ricard-Blum S
2020-05-29	Modified	Cook J

Entities found in this pathway (2)

Input	UniProt Id	Input	UniProt Id
COL1A2	P08123	LAMA3	Q16787

7. ECM proteoglycans (R-HSA-3000178)

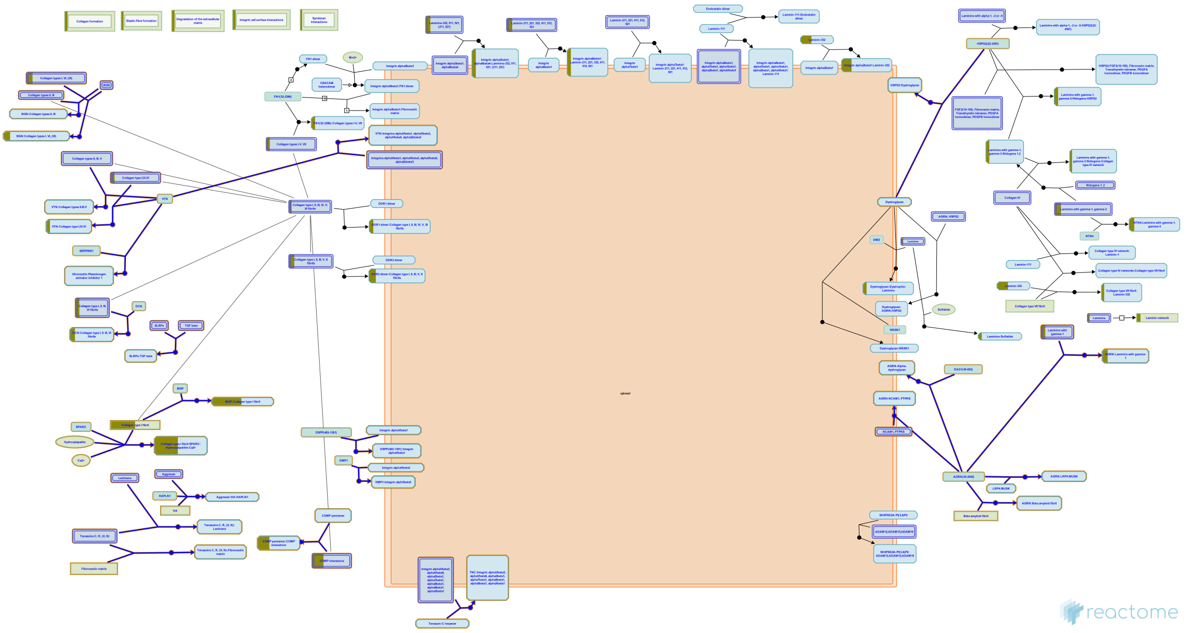

Proteoglycans are major components of the extracellular matrix. In cartilage the matrix constitutes more than 90% of tissue dry weight. Proteoglycans are proteins substituted with glycosaminoglycans (GAGs), linear polysaccharides consisting of a repeating disaccharide, generally of an acetylated amino sugar alternating

with a uronic acid. Most proteoglycans are located in the extracellular

space. Proteoglycans are highly diverse, both in terms of the core proteins and the subtypes of GAG chains, namely chondroitin sulfate (CS), keratan sulfate (KS), dermatan sulfate (DS) and heparan sulfate (HS). Hyaluronan is a non-sulfated GAG whose molecular weight runs into millions of Dalton; in articular cartilage, a single hyaluronan molecule can hold upto 100 aggrecan molecules and these aggregates are stabilized by a link protein.

References

Esko JD, Esko JD, Kimata K, Lindahl U, Varki A, Cummings RD, ... Etzler ME (2009). *Proteoglycans and Sulfated Glycosaminoglycans* .

Kim SH, Turnbull J & Guimond S (2011). Extracellular matrix and cell signalling: the dynamic co-operation of integrin, proteoglycan and growth factor receptor. J. Endocrinol., 209, 139-51.

Hay E (1991). *Cell Biology of Extracellular Matrix* .

Hay E (1991). *Proteoglycans: structure and function, Cell Biology of Extracellular Matrix* .

Edit history

Date	Action	Author
2013-01-10	Authored	Jupe S
2013-01-24	Created	Jupe S
2013-04-26	Edited	Jupe S
2013-05-21	Reviewed	Venkatesan N
2013-05-22	Reviewed	Ricard-Blum S

Date	Action	Author
2020-05-29	Modified	Cook J

Entities found in this pathway (2)

Input	UniProt Id	Input	UniProt Id
COL1A2	P08123	LAMA3	Q16787

8. Signaling by MET (R-HSA-6806834)

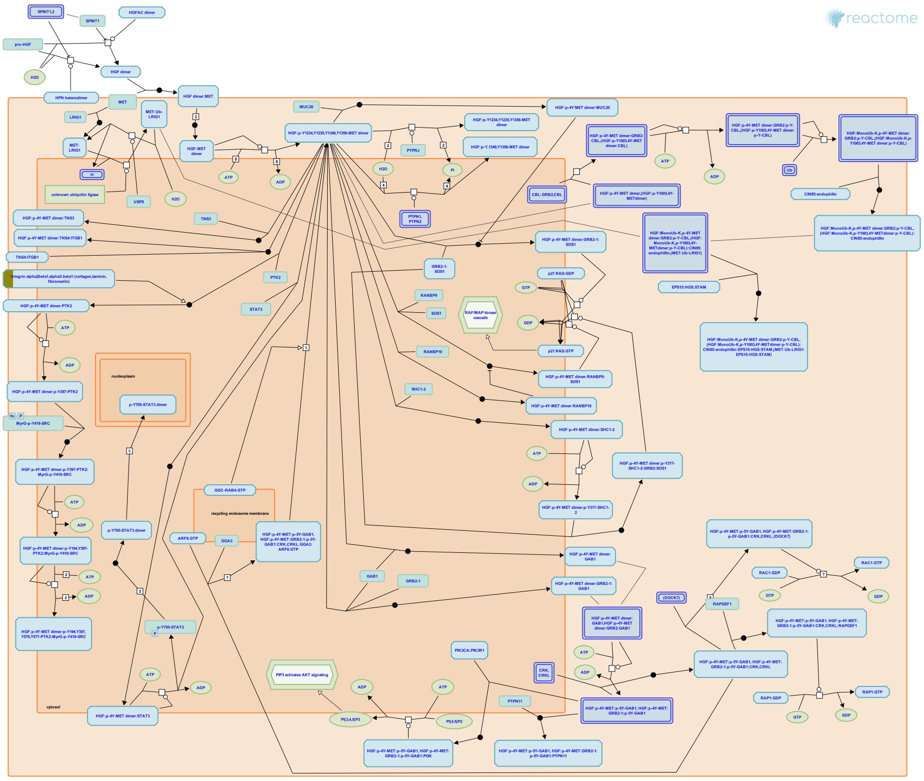

MET is a receptor tyrosine kinase (RTK) (Cooper et al. 1984, Park et al. 1984) activated by binding to its ligand, Hepatocyte growth factor/Scatter factor (HGF/SF) (Bottaro et al. 1991, Naldini et al. 1991). Similar to other related RTKs, such as EGFR, ligand binding induces MET dimerization and trans-autophosphorylation, resulting in the active MET receptor complex (Ferracini et al. 1991, Longati et al. 1994, Rodrigues and Park 1994, Kirchhofer et al. 2004, Stamos et al. 2004, Hays and Watowich 2004). Phosphorylated tyrosines in the cytoplasmic tail of MET serve as docking sites for binding of adapter proteins, such as GRB2, SHC1 and GAB1, which trigger signal transduction cascades that activate PI3K/AKT, RAS, STAT3, PTK2, RAC1 and RAP1 signaling (Ponzetto et al. 1994, Pelicci et al. 1995, Weidner et al. 1995, Besser et al. 1997, Shen and Novak 1997, Beviglia and Kramer 1999, Rodrigues et al. 2000, Sakkab et al. 2000, Schaeper et al. 2000, Lamorte et al. 2002, Wang et al. 2002, Chen and Chen 2006, Palamidessi et al. 2008, Chen et al. 2011, Murray et al. 2014).

Activation of PLC gamma 1 (PLCG1) signaling by MET remains unclear. It has been reported that PLCG1 can bind to MET directly (Ponzetto et al. 1994) or be recruited by phosphorylated GAB1 (Gual et al. 2000). Tyrosine residue Y307 of GAB1 that serves as docking sites for PLCG1 may be phosphorylated either by activated MET (Watanabe et al. 2006) or SRC (Chan et al. 2010). Another PCLG1 docking site on GAB1, tyrosine residue Y373, was reported as the SRC target, while the kinase for the main PLCG1 docking site, Y407 of GAB1, is not known (Chan et al. 2010).

Signaling by MET promotes cell growth, cell survival and motility, which are essential for embryonic development (Weidner et al. 1993, Schmidt et al. 1995, Uehara et al. 1995, Bladt et al. 1995, Maina et al. 1997, Maina et al. 2001, Helmbacher et al. 2003) and tissue regeneration (Huh et al. 2004, Borowiak et al. 2004, Liu 2004, Chmielowiec et al. 2007). MET signaling is frequently aberrantly activated in cancer, through MET overexpression or activating MET mutations (Schmidt et al. 1997, Pennacchietti et al. 2003, Smolen et al. 2006, Bertotti et al. 2009).

Considerable progress has recently been made in the development of HGF-MET inhibitors in cancer therapy. These include inhibitors of HGF activators, HGF inhibitors and MET antagonists, which are protein therapeutics that act outside the cell. Kinase inhibitors function inside the cell and have constituted the largest effort towards MET-based therapeutics (Gherardi et al. 2012).

Pathogenic bacteria of the species Listeria monocytogenes, exploit MET receptor as an entryway to host cells (Shen et al. 2000, Veiga and Cossart 2005, Neimann et al. 2007).

For review of MET signaling, please refer to Birchmeier et al. 2003, Trusolino et al. 2010, Gherardi et al. 2012, Petrini 2015.

References

Ponzetto C, Bardelli A, Zhen Z, Maina F, dalla Zonca P, Giordano S, ... Comoglio PM (1994). A multifunctional docking site mediates signaling and transformation by the hepatocyte growth factor/scatter factor receptor family. Cell, 77, 261-71.

Gual P, Giordano S, Williams TA, Rocchi S, Van Obberghen E & Comoglio PM (2000). Sustained recruitment of phospholipase C-gamma to Gab1 is required for HGF-induced branching tubulogenesis. Oncogene, 19, 1509-18.

Chan PC, Sudhakar JN, Lai CC & Chen HC (2010). Differential phosphorylation of the docking protein Gab1 by c-Src and the hepatocyte growth factor receptor regulates different aspects of cell functions. Oncogene, 29, 698-710.

Ferracini R, Longati P, Naldini L, Vigna E & Comoglio PM (1991). Identification of the major autophosphorylation site of the Met/hepatocyte growth factor receptor tyrosine kinase. J. Biol. Chem., 266, 19558-64.

Longati P, Bardelli A, Ponzetto C, Naldini L & Comoglio PM (1994). Tyrosines1234-1235 are critical for activation of the tyrosine kinase encoded by the MET proto-oncogene (HGF receptor). Oncogene, 9, 49-57.

Edit history

Date	Action	Author
2015-10-27	Created	Orlic-Milacic M
2016-06-14	Edited	Orlic-Milacic M
2016-06-14	Authored	Orlic-Milacic M
2016-07-11	Reviewed	Heynen G, Birchmeier W
2020-05-29	Modified	Cook J

Entities found in this pathway (2)

Input	UniProt Id	Input	UniProt Id
COL1A2	P08123	LAMA3	Q16787

9. RAB GEFs exchange GTP for GDP on RABs (R-HSA-8876198)

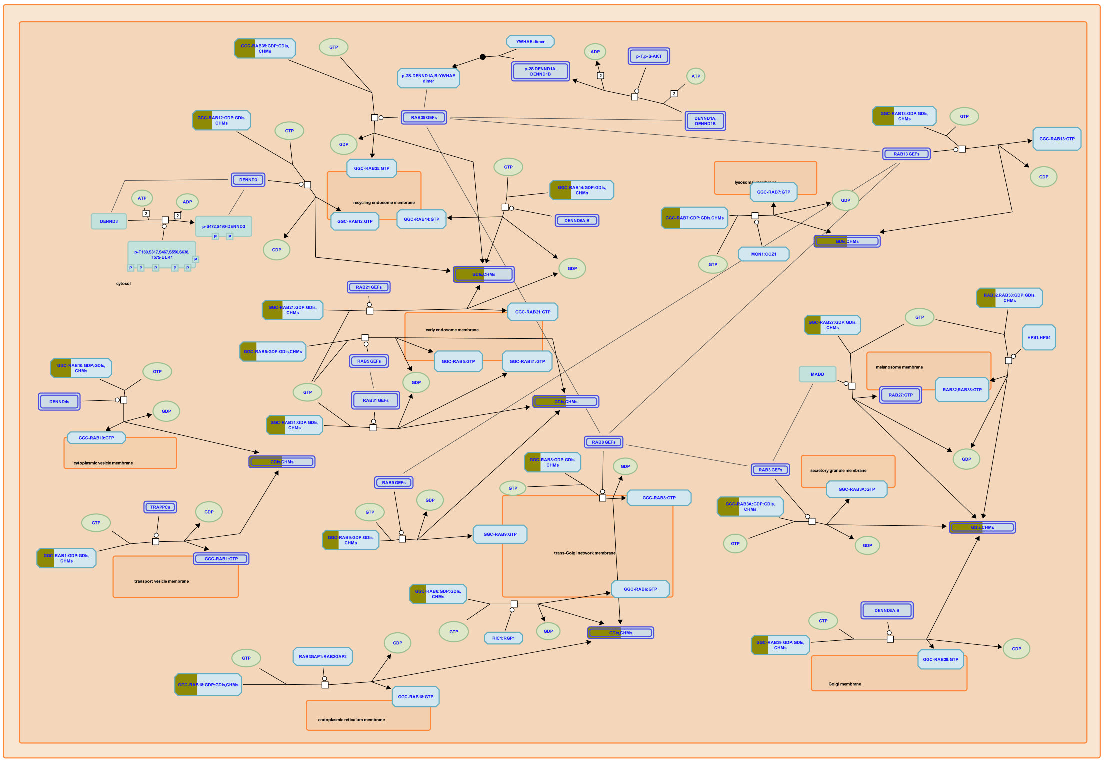

Human cells have more than 60 RAB proteins that are key regulators of intracellular membrane trafficking. These small GTPases contribute to trafficking specificity by localizing to the membranes of different organelles and interacting with effectors such as sorting adaptors, tethering factors, kinases, phosphatases and tubular-vesicular cargo (reviewed in Stenmark et al, 2009; Wandinger-Ness and Zerial, 2014; Zhen and Stenmark, 2015).

RAB localization depends on a number of factors including C-terminal prenylation, the sequence of upstream hypervariable regions and what nucleotide is bound, as well as interaction with RAB-interacting proteins (Chavrier et al, 1991; Ullrich et al, 1993; Soldati et al, 1994; Farnsworth et al, 1994; Seabra, 1996; Wu et al, 2010; reviewed in Stenmark, 2009; Wandinger-Ness and Zerial, 2014). More recently, the activity of RAB GEFs has also been implicated in regulating the localization of RAB proteins (Blumer et al, 2103; Schoebel et al, 2009; Cabrera and Ungermann, 2013; reviewed in Barr, 2013; Zhen and Stenmark, 2015)

In the active, GTP-bound form, RAB proteins are membrane-associated, while in the inactive GDP-bound form, RABs are extracted from the target membrane and exist in a soluble form in complex with GDP dissociation inhibitors (GDIs) (Ullrich et al, 1993; Soldati et al, 1994; Gavriljuk et al, 2013). Conversion between the inactive and active form relies on the activities of RAB guanine nucleotide exchange factors (GEFs) and GTPase activating proteins (GAPs) (Yoshimura et al, 2010; Wu et al, 2011; Pan et al, 2006; Frasa et al, 2012; reviewed in Stenmark, 2009; Wandinger-Ness and Zerial, 2014; Ishida et al, 2016).

Newly synthesized RABs are bound to a RAB escort protein, CHM (also known as REP1) or CHML (REP2) (Alexandrov et al, 1994; Shen and Seabra, 1996). CHM/REP proteins are the substrate-binding component of the trimeric RAB geranylgeranyltransferase enzyme (GGTaseII) along with the two catalytic subunits RABGGTA and RABGGTB (reviewed in Gutkowska and Swiezewska, 2012; Palsuledesai and Distefano, 2015). REP proteins recruit the unmodified RAB in its GDP-bound state to the GGTase for sequential geranylgeranylation at one or two C-terminal cysteine residues (Alexandrov et al, 1994; Seabra et al 1996; Shen and Seabra, 1996; Baron and Seabra, 2008). After geranylation, CHM/REP proteins remain in complex with the geranylated RAB and escort it to its target membrane, where RAB activity is regulated by GAPs, GEFs, GDIs and membrane-bound GDI displacement factors (GDFs) (Sivars et al, 2003; reviewed in Stenmark, 2009; Wandinger-Ness and Zerial, 2014).

Unlike the RAB GAPS, which (to date) all contain a shared TBC domain, RAB GEFs are structurally diverse and range from monomeric to multisubunit complexes (reviewed in Fukuda et al, 2011; Frasa et al, 2012; Cherfils and Zeghouf, 2013; Ishida et al, 2016). While many GEFs contain one of three conserved GEF domains identified to date - the DENN (differentially expressed in normal and neoplastic cell) domain, the VPS9 domain and the SEC2 domain- other GEFs lack a conserved domain (reviewed in Ishida et al, 2016). Based on sequence conservation and subunit organization, GEFs can be grouped into 6 general classes: the DENND-containing GEFs, the VPS9-containing GEFs (both monomeric), the SEC2-containing GEFs (homodimeric), heterodimeric GEF complexes such as RIC1:RGP1, the multisubunit TRAPPC GEF, and others (reviewed in Barr and Lambright, 2010; Marat et al, 2011; Ishida et al, 2016). GEFs for many RABs have still not been identified, however.

References

Stenmark H (2009). Rab GTPases as coordinators of vesicle traffic. Nat. Rev. Mol. Cell Biol., 10, 513-25.

Wandinger-Ness A & Zerial M (2014). Rab proteins and the compartmentalization of the endosomal system. Cold Spring Harb Perspect Biol, 6, a022616.

Chavrier P, Gorvel JP, Stelzer E, Simons K, Gruenberg J & Zerial M (1991). Hypervariable C-terminal domain of rab proteins acts as a targeting signal. Nature, 353, 769-72.

Ullrich O, Stenmark H, Alexandrov K, Huber LA, Kaibuchi K, Sasaki T, ... Zerial M (1993). Rab GDP dissociation inhibitor as a general regulator for the membrane association of rab proteins. J. Biol. Chem., 268, 18143-50.

Soldati T, Shapiro AD, Svejstrup AB & Pfeffer SR (1994). Membrane targeting of the small GTPase Rab9 is accompanied by nucleotide exchange. Nature, 369, 76-8.

Edit history

Date	Action	Author
2016-06-10	Created	Rothfels K
2016-06-29	Edited	Rothfels K
2016-06-29	Authored	Rothfels K
2016-08-03	Reviewed	Marat AL
2020-05-29	Modified	Cook J

Entities found in this pathway (1)

Input	UniProt Id
GDI1	P31150, P50395

10. Collagen formation (R-HSA-1474290)

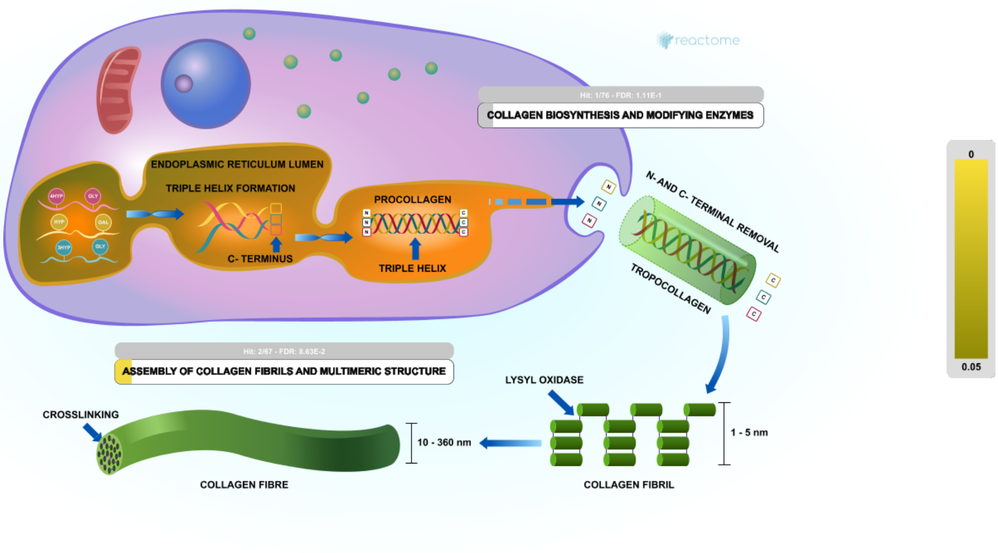

Collagen is a family of at least 29 structural proteins derived from over 40 human genes (Myllyharju & Kivirikko 2004). It is the main component of connective tissue, and the most abundant protein in mammals making up about 25% to 35% of whole-body protein content. A defining feature of collagens is the formation of trimeric left-handed polyproline II-type helical collagenous regions. The packing within these regions is made possible by the presence of the smallest amino acid, glycine, at every third residue, resulting in a repeating motif Gly-X-Y where X is often proline (Pro) and Y often 4-hydroxyproline (4Hyp). Gly-Pro-Hyp is the most common triplet in collagen (Ramshaw et al. 1998). Collagen peptide chains also have non-collagenous domains, with collagen subclasses having common chain structures. Collagen fibrils are mostly found in fibrous tissues such as tendon, ligament and skin. Other forms of collagen are abundant in cornea, cartilage, bone, blood vessels, the gut, and intervertebral disc. In muscle tissue, collagen is a major component of the endomysium, constituting up to 6% of muscle mass. Gelatin, used in food and industry, is collagen that has been irreversibly hydrolyzed.

On the basis of their fibre architecture in tissues, the genetically distinct collagens have been divided into subgroups. Group 1 collagens have uninterrupted triple-helical domains of about 300 nm, forming large extracellular fibrils. They are referred to as the fibril-forming collagens, consisting of collagens types I, II, III, V, XI, XXIV and XXVII. Group 2 collagens are types IV and VII, which have extended triple helices (>350 nm) with imperfections in the Gly-X-Y repeat sequences. Group 3 are the short-chain collagens. These have two subgroups. Group 3A have continuous triple-helical domains (type VI, VIII and X). Group 3B have interrupted triple-helical domains, referred to as the fibril-associated collagens with interrupted triple helices (FACIT collagens, Shaw & Olsen 1991). FACITs include collagen IX, XII, XIV, XVI, XIX, XX, XXI, XXII and XXVI plus the transmembrane collagens (XIII, XVII, XXIII and XXV) and the multiple triple helix domains and interruptions (Multiplexin) collagens XV and XVIII (Myllyharju & Kivirikko 2004). The non-collagenous domains of collagens have regulatory functions; several are biologically active when cleaved from the main peptide chain. Fibrillar collagen peptides all have a large triple helical domain (COL1) bordered by N and C terminal extensions, called the N- and C-propeptides, which are cleaved prior to formation of the collagen fibril. The intact form is referred to as a collagen propeptide, not procollagen, which is used to refer to the trimeric triple-helical precursor of collagen before the propeptides are removed. The C-propeptide, also called the NC1 domain, directs chain association during assembly of the procollagen molecule from its three constituent alpha chains (Hulmes 2002).

Fibril forming collagens are the most familiar and best studied subgroup. Collagen fibres are aggregates or bundles of collagen fibrils, which are themselves polymers of tropocollagen complexes, each consisting of three polypeptide chains known as alpha chains. Tropocollagens are considered the subunit of larger collagen structures. They are approximately 300 nm long and 1.5 nm in diameter, with a left-handed triple-helical structure, which becomes twisted into a right-handed coiled-coil 'super helix' in the collagen fibril. Tropocollagens in the extracellular space polymerize spontaneously with regularly staggered ends (Hulmes 2002). In fibrillar collagens the molecules are staggered by about 67 nm, a unit known as D that changes depending upon the hydration state. Each D-period contains slightly more than four collagen molecules so that every D-period repeat of the microfibril has a region containing five molecules in cross-section, called the 'overlap', and a region containing only four molecules, called the 'gap'. The triple-helices are arranged in a hexagonal or quasi-hexagonal array in cross-section, in both the gap and overlap regions (Orgel et al. 2006). Collagen molecules cross-link covalently to each other via lysine and hydroxylysine side chains. These cross-links are unusual, occuring only in collagen and elastin, a related protein.

The macromolecular structures of collagen are diverse. Several group 3 collagens associate with larger collagen fibers, serving as molecular bridges which stabilize the organization of the extracellular matrix. Type IV collagen is arranged in an interlacing network within the dermal-epidermal junction and vascular basement membranes. Type VI collagen forms distinct microfibrils called beaded filaments. Type VII collagen forms anchoring fibrils. Type VIII and X collagens form hexagonal networks. Type XVII collagen is a component of hemidesmosomes where it is complexed wtih alpha6Beta4 integrin, plectin, and laminin-332 (de Pereda et al. 2009). Type XXIX collagen has been recently reported to be a putative epidermal collagen with highest expression in suprabasal layers (Soderhall et al. 2007). Collagen fibrils/aggregates arranged in varying combinations and concentrations in different tissues provide specific tissue properties. In bone, collagen triple helices lie in a parallel, staggered array with 40 nm gaps between the ends of the tropocollagen subunits, which probably serve as nucleation sites for the deposition of crystals of the mineral component, hydroxyapatite (Ca10(PO4)6(OH)2) with some phosphate. Collagen structure affects cell-cell and cell-matrix communication, tissue construction in growth and repair, and is changed in development and disease (Sweeney et al. 2006, Twardowski et al. 2007). A single collagen fibril can be heterogeneous along its axis, with significantly different mechanical properties in the gap and overlap regions, correlating with the different molecular organizations in these regions (Minary-Jolandan & Yu 2009).

References

Prockop DJ & Kivirikko KI (1995). Collagens: molecular biology, diseases, and potentials for therapy . Annu Rev Biochem, 64, 403-34.

Gordon MK & Hahn RA (2010). Collagens. Cell Tissue Res, 339, 247-57.

Edit history

Date	Action	Author
2011-08-05	Authored	Jupe S
2011-08-05	Created	Jupe S
2012-04-11	Edited	Jupe S
2012-05-24	Reviewed	Canty-Laird EG
2020-05-29	Modified	Cook J

Entities found in this pathway (2)

Input	UniProt Id	Input	UniProt Id
COL1A2	P08123	LAMA3	Q16787

11. Extracellular matrix organization (R-HSA-1474244)

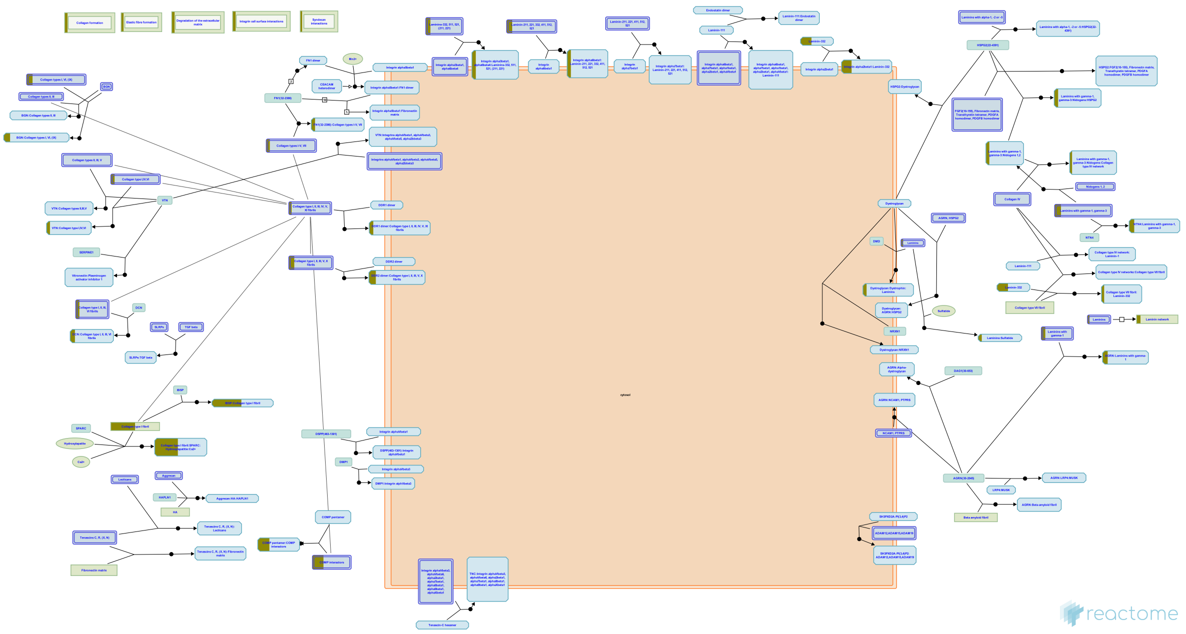

The extracellular matrix is a component of all mammalian tissues, a network consisting largely of the fibrous proteins collagen, elastin and associated-microfibrils, fibronectin and laminins embedded in a viscoelastic gel of anionic proteoglycan polymers. It performs many functions in addition to its structural role; as a major component of the cellular microenvironment it influences cell behaviours such as proliferation, adhesion and migration, and regulates cell differentiation and death (Hynes 2009).

ECM composition is highly heterogeneous and dynamic, being constantly remodeled (Frantz et al. 2010) and modulated, largely by matrix metalloproteinases (MMPs) and growth factors that bind to the ECM influencing the synthesis, crosslinking and degradation of ECM components (Hynes 2009). ECM remodeling is involved in the regulation of cell differentiation processes such as the establishment and maintenance of stem cell niches, branching morphogenesis, angiogenesis, bone remodeling, and wound repair. Redundant mechanisms modulate the expression and function of ECM modifying enzymes. Abnormal ECM dynamics can lead to deregulated cell proliferation and invasion, failure of cell death, and loss of cell differentiation, resulting in congenital defects and pathological processes including tissue fibrosis and cancer.

Collagen is the most abundant fibrous protein within the ECM constituting up to 30% of total protein in multicellular animals. Collagen provides tensile strength. It associates with elastic fibres, composed of elastin and fibrillin microfibrils, which give tissues the ability to recover after stretching. Other ECM proteins such as fibronectin, laminins, and matricellular proteins participate as connectors or linking proteins (Daley et al. 2008).

Chondroitin sulfate, dermatan sulfate and keratan sulfate proteoglycans are structural components associated with collagen fibrils (Scott & Haigh 1985; Scott & Orford 1981), serving to tether the fibril to the surrounding matrix. Decorin belongs to the small leucine-rich repeat proteoglycan family (SLRPs) which also includes biglycan, fibromodulin, lumican and asporin. All appear to be involved in collagen fibril formation and matrix assembly (Ameye & Young 2002).

ECM proteins such as osteonectin (SPARC), osteopontin and thrombospondins -1 and -2, collectively referred to as matricellular proteins (reviewed in Mosher & Adams 2012) appear to modulate cell-matrix interactions. In general they induce de-adhesion, characterized by disruption of focal adhesions and a reorganization of actin stress fibers (Bornstein 2009). Thrombospondin (TS)-1 and -2 bind MMP2. The resulting complex is endocytosed by the low-density lipoprotein receptor-related protein (LRP), clearing MMP2 from the ECM (Yang et al. 2001).

Osteopontin (SPP1, bone sialoprotein-1) interacts with collagen and fibronectin (Mukherjee et al. 1995). It also contains several cell adhesive domains that interact with integrins and CD44.

Aggrecan is the predominant ECM proteoglycan in cartilage (Hardingham & Fosang 1992). Its relatives include versican, neurocan and brevican (Iozzo 1998). In articular cartilage the major non-fibrous macromolecules are aggrecan, hyaluronan and hyaluronan and proteoglycan link protein 1 (HAPLN1). The high negative charge density of these molecules leads to the binding of large amounts of water (Bruckner 2006). Hyaluronan is bound by several large proteoglycans proteoglycans belonging to the hyalectan family that form high-molecular weight aggregates (Roughley 2006), accounting for the turgid nature of cartilage.

The most significant enzymes in ECM remodeling are the Matrix Metalloproteinase (MMP) and A disintegrin and metalloproteinase with thrombospondin motifs (ADAMTS) families (Cawston & Young 2010). Other notable ECM degrading enzymes include plasmin and cathepsin G. Many ECM proteinases are initially present as precursors, activated by proteolytic processing. MMP precursors include an amino prodomain which masks the catalytic Zn-binding motif (Page-McCawet al. 2007). This can be removed by other proteinases, often other MMPs. ECM proteinases can be inactivated by degradation, or blocked by inhibitors. Some of these inhibitors, including alpha2-macroglobulin, alpha1-proteinase inhibitor, and alpha1-chymotrypsin can inhibit a large variety of proteinases (Woessner & Nagase 2000). The tissue inhibitors of metalloproteinases (TIMPs) are potent MMP inhibitors (Brew & Nagase 2010).

References

Frantz C, Stewart KM & Weaver VM (2010). The extracellular matrix at a glance. J Cell Sci, 123, 4195-200.

Lu P, Takai K, Weaver VM & Werb Z (2011). Extracellular matrix degradation and remodeling in development and disease. Cold Spring Harb Perspect Biol, 3.

Bosman FT & Stamenkovic I (2003). Functional structure and composition of the extracellular matrix. J Pathol, 200, 423-8.

Edit history

Date	Action	Author
2011-08-05	Created	Jupe S
2011-09-09	Authored	Jupe S
2012-02-21	Edited	Jupe S
2012-02-28	Reviewed	D'Eustachio P
2013-05-21	Reviewed	Venkatesan N
2013-05-22	Reviewed	Ricard-Blum S

Date	Action	Author
2020-05-29	Modified	Cook J

Entities found in this pathway (3)

Input	UniProt Id	Input	UniProt Id	Input	UniProt Id
COL1A2	P08123	EMILIN1	Q9Y6C2	LAMA3	Q16787

12. Rab regulation of trafficking (R-HSA-9007101)

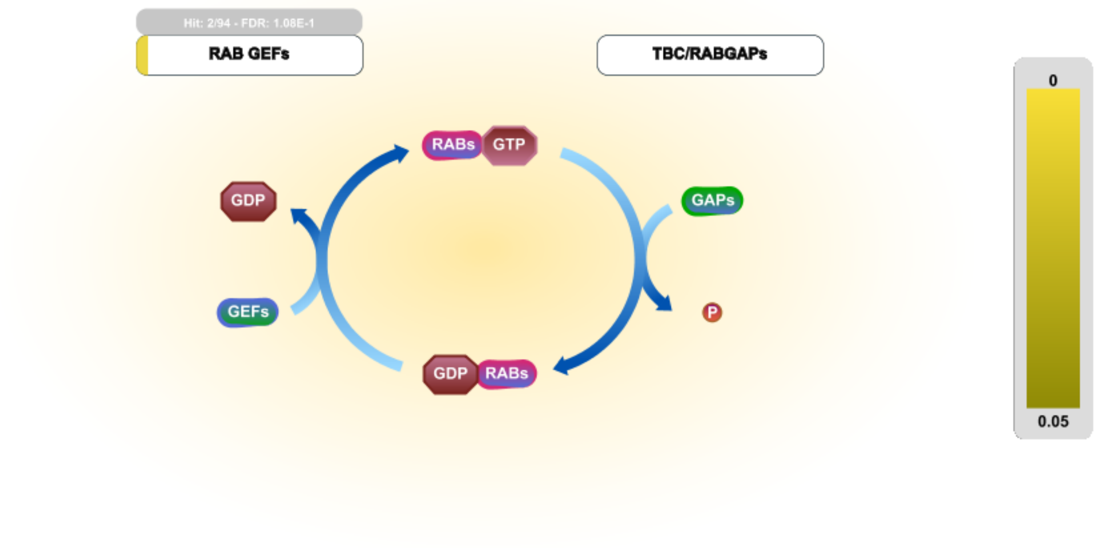

Human cells have more than 60 RAB proteins that are key regulators of intracellular membrane trafficking. These small GTPases contribute to trafficking specificity by localizing to the membranes of different organelles and interacting with effectors such as sorting adaptors, tethering factors, kinases, phosphatases and tubular-vesicular cargo (reviewed in Stenmark et al, 2009; Wandinger-Ness and Zerial, 2014; Zhen and Stenmark, 2015).

RAB localization depends on a number of factors including C-terminal prenylation, the sequence of upstream hypervariable regions and what nucleotide is bound, as well as interaction with RAB-interacting proteins (Chavrier et al, 1991; Ullrich et al, 1993; Soldati et al, 1994; Farnsworth et al, 1994; Seabra, 1996; Wu et al, 2010; reviewed in Stenmark, 2009; Wandinger-Ness and Zerial, 2014). More recently, the activity of RAB GEFs has also been implicated in regulating the localization of RAB proteins (Blumer et al, 2103; Schoebel et al, 2009; Cabrera and Ungermann, 2013; reviewed in Barr, 2013; Zhen and Stenmark, 2015).

References

Barr FA & Lambright DG (2010). Rab GEFs and GAPs. Curr. Opin. Cell Biol., 22, 461-70.

Stenmark H (2009). Rab GTPases as coordinators of vesicle traffic. Nat. Rev. Mol. Cell Biol., 10, 513-25.

Edit history

Date	Action	Author
2016-06-29	Authored	Rothfels K
2016-08-03	Reviewed	Marat AL
2017-05-26	Edited	Jupe S
2017-05-26	Created	Jupe S

Date	Action	Author
2020-05-29	Modified	Cook J

Entities found in this pathway (1)

Input	UniProt Id
GDI1	P31150, P50395

13. Type I hemidesmosome assembly (R-HSA-446107)

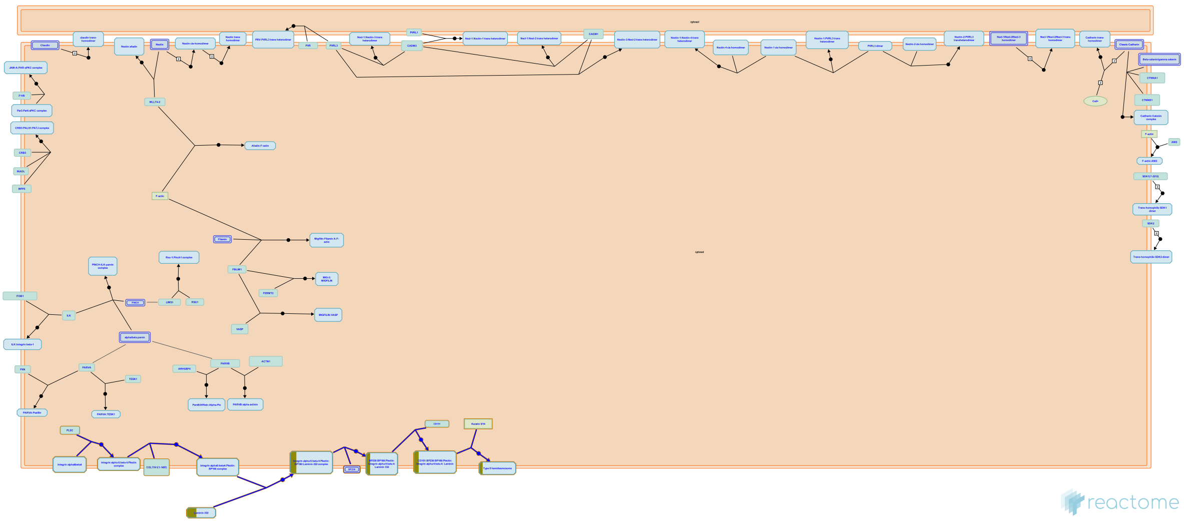

Hemidesmosomes (HDs) are specialized multiprotein junctional complexes that connect the keratin cytoskeleton of epithelial cells to the extracellular matrix and play a critical role in the maintenance of tissue structure and integrity (reviewed in Litjens et al., 2006). HDs mediate adhesion of epithelial cells to the underlying basement membrane in stratified squamous, transitional and pseudostratified epithelia (Jones et al., 1994 ; Borradori and Sonnenberg, 1996). Classical Type I HDs are found in stratified and pseudo-stratified epithelia, such as the skin, and contain a6b4, plectin, tetraspanin CD151 and the bullous pemphigoid (BP) antigens BP180 and BP230 (reviewed in Litjens et al., 2006). While HDs function in promoting stable adhesion, they are highly dynamic structures that are able to disassemble quickly, for example, during cell division, differentiation, or migration (see Margadant et al, 2008).

References

Litjens SH, de Pereda JM & Sonnenberg A (2006). Current insights into the formation and breakdown of hemidesmosomes. Trends Cell Biol, 16, 376-83.

Edit history

Date	Action	Author
2009-11-04	Edited	Matthews L
2009-11-04	Authored	Matthews L
2009-11-09	Created	Matthews L
2009-11-15	Reviewed	Sonnenberg A
2020-05-29	Modified	Cook J

Entities found in this pathway (1)

Input	UniProt Id
LAMA3	Q16787

14. AMPK inhibits chREBP transcriptional activation activity (R-HSA-163680)

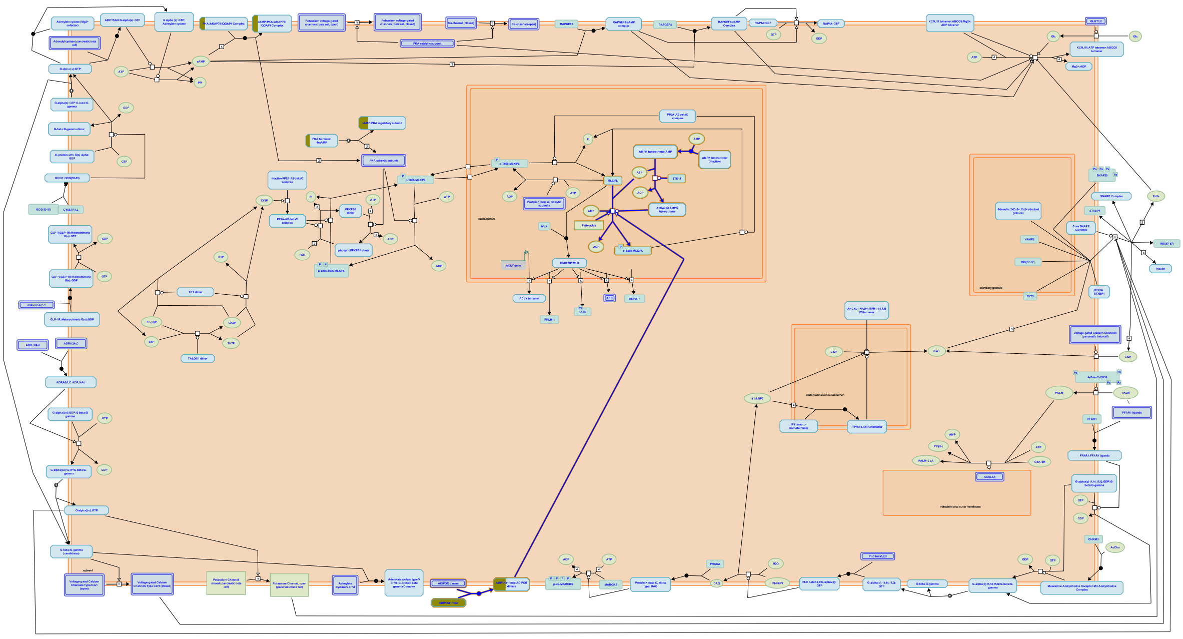

Cellular compartments: nucleoplasm.

AMP-activated protein kinase (AMPK) is a sensor of cellular energy levels. A high cellular ratio of AMP:ATP triggers the phosphorylation and activation of AMPK. Activated AMPK in turn phosphorylates a wide array of target proteins, as shown in the figure below (reproduced from (Hardie et al. 2003), with the permission of D.G. Hardie). These targets include ChREBP (Carbohydrate Response Element Binding Protein), whose inactivation by phosphorylation reduces transcription of key enzymes of the glycolytic and lipogenic pathways.

References

Kawaguchi T, Osatomi K, Yamashita H, Kabashima T & Uyeda K (2002). Mechanism for fatty acid sparing effect on glucose-induced transcription: regulation of carbohydrate-responsive element-binding protein by AMP-activated protein kinase. J Biol Chem, 277, 3829-35.

Cheung PC, Salt IP, Davies SP, Hardie DG & Carling D (2000). Characterization of AMP-activated protein kinase gamma-subunit isoforms and their role in AMP binding. Biochem J, 346, 659-69.

Hardie DG, Scott JW, Pan DA & Hudson ER (2003). Management of cellular energy by the AMP-activated protein kinase system. FEBS Lett, 546, 113-20.

Hardie DG (2004). The AMP-activated protein kinase pathway--new players upstream and downstream. J Cell Sci, 117, 5479-87.

Edit history

Date	Action	Author
2005-05-05	Created	Gopinathrao G
2005-05-13	Authored	Gopinathrao G
2020-05-29	Modified	Cook J

Entities found in this pathway (1)

Input	UniProt Id
EIF4A2	Q15848

15. GP1b-IX-V activation signalling (R-HSA-430116)

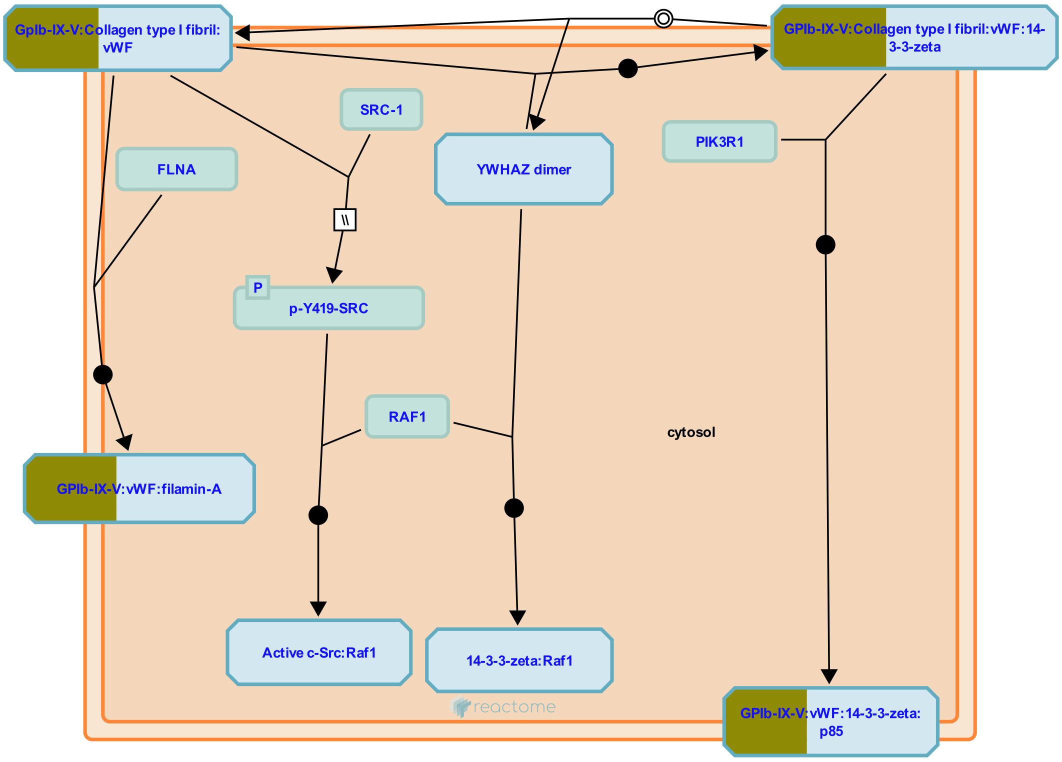

Cellular compartments: plasma membrane.

The platelet GPIb complex (GP1b-IX-V) together with GPVI are primarily responsible for regulating the initial adhesion of platelets to the damaged blood vessel and platelet activation. The importance of GPIb is demonstrated by the bleeding problems in patients with Bernard-Soulier syndrome where this receptor is either absent or defective. GP1b-IX-V binds von Willebrand Factor (vWF) to resting platelets, particularly under conditions of high shear stress. This transient interaction is the first stage of the vascular repair process. Activation of GP1b-IX-V on exposure of the fibrous matrix following atherosclerotic plaque rupture, or in occluded arteries, is a major contributory factor leading to thrombus formation leading to heart attack or stroke.
GpIb also binds thrombin (Yamamoto et al. 1986), at a site distinct from the site of vWF binding, acting as a docking site for thrombin which then activates Proteinase Activated Receptors leading to enhanced platelet activation (Dormann et al. 2000).

References

Andrews RK & Berndt MC (2004). Platelet physiology and thrombosis. Thromb Res, 114, 447-53.

Ruggeri ZM & Mendolicchio GL (2007). Adhesion mechanisms in platelet function. Circ Res, 100, 1673-85.

Edit history

Date	Action	Author
2009-06-03	Authored	Akkerman JW
2009-07-30	Created	Jupe S
2010-06-07	Edited	Jupe S

Date	Action	Author
2010-06-07	Reviewed	Kunapuli SP
2020-05-29	Modified	Cook J

Entities found in this pathway (1)

Input	UniProt Id
COL1A2	P08123

16. Rho GTPase cycle (R-HSA-194840)

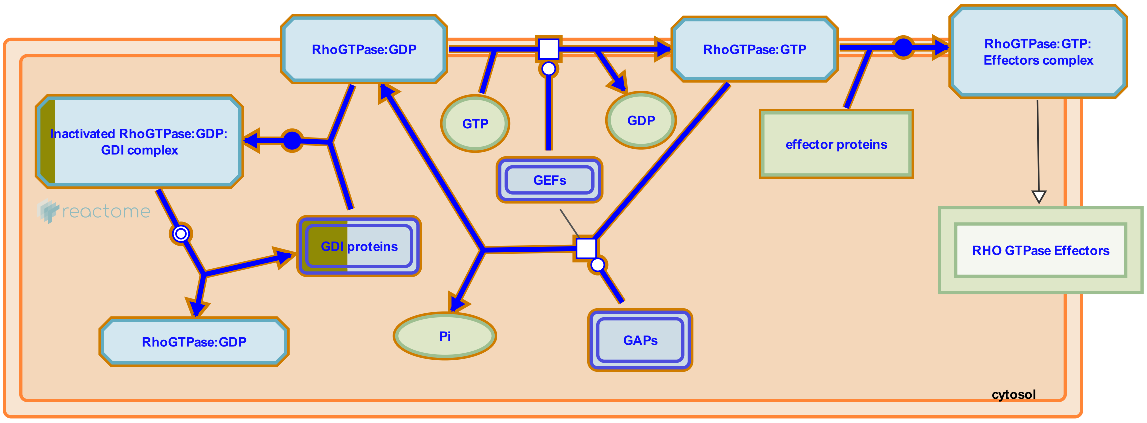

Cellular compartments: cytosol.

The cycling of Rho GTPases is tightly controlled by three classes of protein. These are (1) guanine nucleotide dissociation inhibitors or GDIs, which maintain Rho proteins in an inactive state in the cytoplasm, (2) guanine nucleotide exchange factors or GEFs, which destabilize the interaction between Rho proteins and their bound nucleotide, the net result of which is the exchange of bound GDP for the more abundant GTP, and (3) GTPase Activating Proteins or GAPs, which stimulate the low intrinsic GTP hydrolysis activity of Rho family members, thus promoting their inactivation. GDIs, GEFs, and GAPs are themselves subject to tight regulation, and the overall level of Rho activity reflects the balance of their activities.

In their active GTP-bound state, Rho family members have the ability to interact with a large variety of so-called effector proteins. By changing the subcellular localization of effectors, by altering their enzymatic properties, or by directing the formation of specific effector complexes, members of the Rho family mediate their various effects.

This Rho GTPase cycle is diagrammed in the figure below. External or internal cues promote the release of Rho GTPases from the inhibitory complex (1) which allows them to associate with the plasma membrane (2) where they are activated by GEFs (3) and can signal to effector proteins. Then, GAPs inactivate the GTPases by accelerating the intrinsic GTPase activity, leading to the GDP bound form (4). Once again, the GDI molecules stabilize the inactive GDP bound form in the cytoplasm, waiting for further instructions (5). (Figure and text from Tcherkezian and Lamarche Vane, 2007).

References

Van Aelst L & D'Souza-Schorey C (1997). Rho GTPases and signaling networks. Genes Dev, 11, 2295-322.

Tcherkezian J & Lamarche-Vane N (2007). Current knowledge of the large RhoGAP family of proteins. Biol Cell, 99, 67-86.

Edit history

Date	Action	Author
2007-03-29	Created	Gopinathrao G
2007-04-03	Edited	Gopinathrao G

Date	Action	Author
2007-04-29	Reviewed	Bernards A
2007-04-29	Authored	Van Aelst L
2020-06-04	Modified	Cook J

Entities found in this pathway (1)

Input	UniProt Id
GDI1	P31150, P50395

17. Integration of energy metabolism (R-HSA-163685)

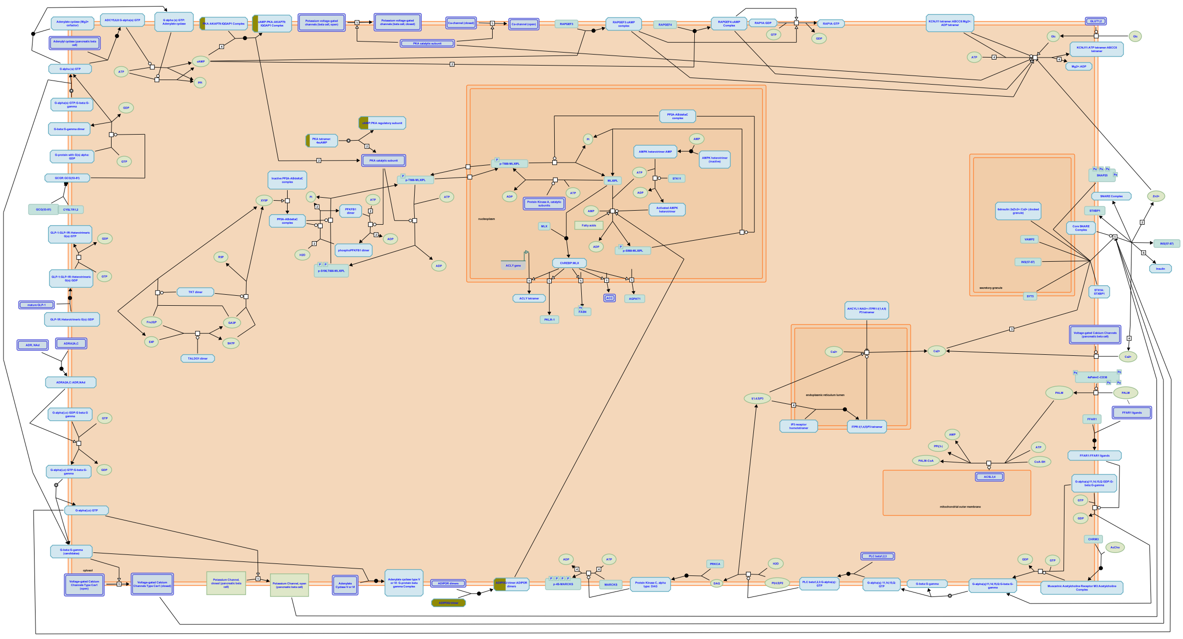

Cellular compartments: cytosol.

Many hormones that affect individual physiological processes including the regulation of appetite, absorption, transport, and oxidation of foodstuffs influence energy metabolism pathways. While **insulin** mediates the storage of excess nutrients, **glucagon** is involved in the mobilization of energy resources in response to low blood glucose levels, principally by stimulating hepatic glucose output. Small doses of glucagon are sufficient to induce significant glucose elevations. These hormone-driven regulatory pathways enable the body to sense and respond to changed amounts of nutrients in the blood and demands for energy.

Glucagon and Insulin act through various metabolites and enzymes that target specific steps in metabolic pathways for sugar and fatty acids. The processes responsible for the long-term control of fat synthesis and short term control of glycolysis by key metabolic products and enzymes are annotated in this module as six specific pathways:

Pathway 1. Glucagon signalling in metabolic pathways: In response to low blood glucose, pancreatic alpha-cells release glucagon. The binding of glucagon to its receptor results in increased cAMP synthesis, and Protein Kinase A (PKA) activation.

Pathway 2. PKA mediated phosphorylation:PKA phosphorylates key enzymes, e.g., 6-Phosphofructo-2-kinase /Fructose-2,6-bisphosphatase (PF2K-Pase) at serine 36, and regulatory proteins, e.g., Carbohydrate Response Element Binding Protein (ChREBP) at serine 196 and threonine 666.

In brief, the binding of insulin to its receptor leads to increased protein phosphatase activity and to hydrolysis of cAMP by cAMP phosphodiesterase. These events counteract the regulatory effects of glucagon.

Pathway 3: Insulin stimulates increased synthesis of Xylulose-5-phosphate (Xy-5-P). Activation of the insulin receptor results indirectly in increased Xy-5-P synthesis from Glyceraldehyde-3-phosphate and Fructose-6-phosphate. Xy-5-P, a metabolite of the pentose phosphate pathway, stimulates protein phosphatase PP2A.

Pathway 4: AMP Kinase (AMPK) mediated response to high AMP:ATP ratio: In response to diet with high fat content or low energy levels, the cytosolic AMP:ATP ratio is increased. AMP triggers a complicated cascade of events. In this module we have annotated only the phosphorylation of ChREBP by AMPK at serine 568, which inactivates this transcription factor.

Pathway 5: Dephosphorylation of key metabolic factors by PP2A: Xy-5-P activated PP2A efficiently dephosphorylates phosphorylated PF2K-Pase resulting in the higher output of F-2,6-P2 that enhances PFK activity in the glycolytic pathway. PP2A also dephosphorylates (and thus activates) cytosolic and nuclear ChREBP.

Pathway 6: Transcriptional activation of metabolic genes by ChREBP: Dephosphorylated ChREBP activates the transcription of genes involved in glucose metabolism such as pyruvate kinase, and lipogenic genes such as acetyl-CoA carboxylase, fatty acid synthetase, acyl CoA synthase and glycerol phosphate acyl transferase.

The illustration below summarizes this network of events. Black lines are metabolic reactions, red lines are negative regulatory events, and green lines are positive regulatory events (figure reused with permission from Veech (2003) - Copyright (2003) National Academy of Sciences, U.S.A.).

References

Veech RL (2003). A humble hexose monophosphate pathway metabolite regulates short- and long-term control of lipogenesis. Proc Natl Acad Sci U S A, 100, 5578-80.

Jiang G & Zhang BB (2003). Glucagon and regulation of glucose metabolism. Am J Physiol Endocrinol Metab, 284, E671-8.

Hardie DG (2004). The AMP-activated protein kinase pathway--new players upstream and downstream. J Cell Sci, 117, 5479-87.

Edit history

Date	Action	Author
2005-05-11	Authored	Gopinathrao G, D'Eustachio P
2005-05-11	Created	Gopinathrao G, D'Eustachio P
2005-09-10	Reviewed	Rush MG
2020-05-29	Modified	Cook J

Entities found in this pathway (2)

Input	UniProt Id	Input	UniProt Id
EIF4A2	Q15848	PRKAR2B	P31323

18. Degradation of the extracellular matrix (R-HSA-1474228)

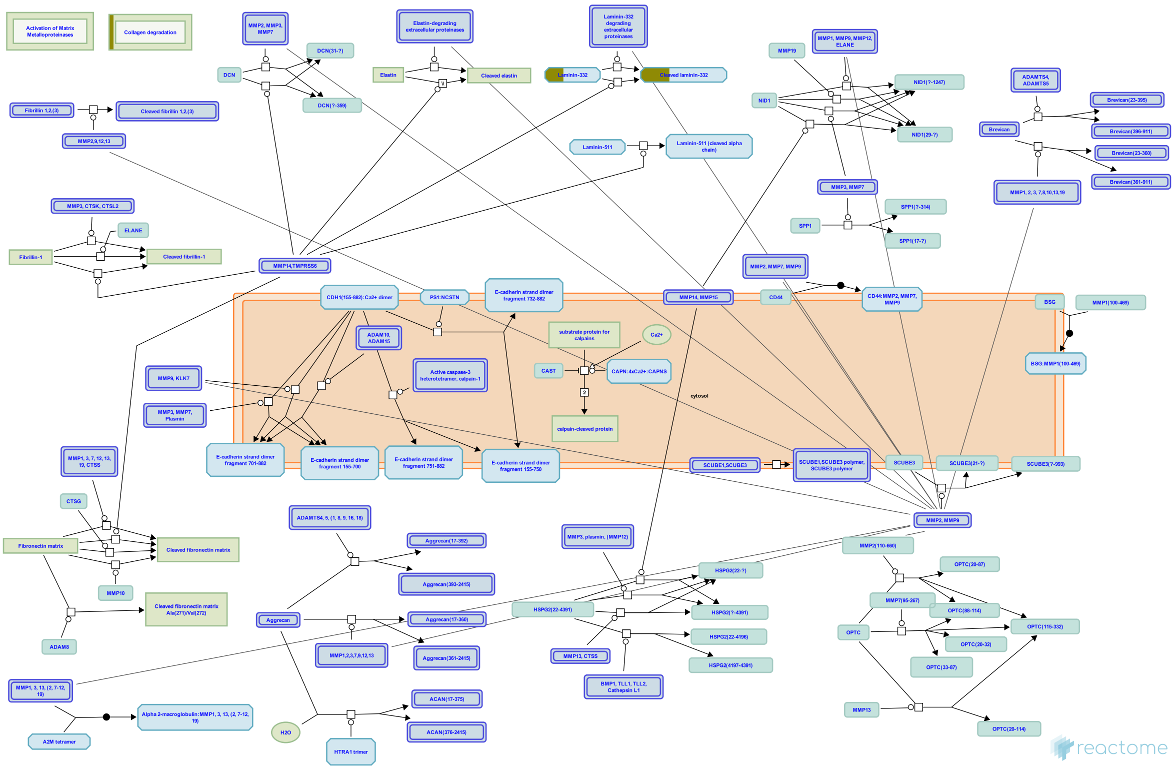

Matrix metalloproteinases (MMPs), previously referred to as matrixins because of their role in degradation of the extracellular matrix (ECM), are zinc and calcium dependent proteases belonging to the metzincin family. They contain a characteristic zinc-binding motif HEXXHXXGXXH (Stocker & Bode 1995) and a conserved Methionine which forms a Met-turn. Humans have 24 MMP genes giving rise to 23 MMP proteins, as MMP23 is encoded by two identical genes. All MMPs contain an N-terminal secretory signal peptide and a prodomain with a conserved PRCGXPD motif that in the inactive enzyme is localized with the catalytic site, the cysteine acting as a fourth unpaired ligand for the catalytic zinc atom. Activation involves delocalization of the domain containing this cysteine by a conformational change or proteolytic cleavage, a mechanism referred to as the cysteine-switch (Van Wart & Birkedal-Hansen 1990). Most MMPs are secreted but the membrane type MT-MMPs are membrane anchored and some MMPs may act on intracellular proteins. Various domains determine substrate specificity, cell localization and activation (Hadler-Olsen et al. 2011). MMPs are regulated by transcription, cellular location (most are not activated until secreted), activating proteinases that can be other MMPs, and by metalloproteinase inhibitors such as the tissue inhibitors of metalloproteinases (TIMPs). MMPs are best known for their role in the degradation and removal of ECM molecules. In addition, cleavage of the ECM and other cell surface molecules can release ECM-bound growth factors, and a number of non-ECM proteins are substrates of MMPs (Nagase et al. 2006). MMPs can be divided into subgroups based on domain structure and substrate specificity but it is clear that these are somewhat artificial, many MMPs belong to more than one functional group (Vise & Nagase 2003, Somerville et al. 2003).

References

Lu P, Takai K, Weaver VM & Werb Z (2011). Extracellular matrix degradation and remodeling in development and disease. Cold Spring Harb Perspect Biol, 3.

Edit history

Date	Action	Author
2011-08-05	Created	Jupe S
2011-09-09	Authored	Jupe S
2012-02-21	Edited	Jupe S
2012-02-28	Reviewed	D'Eustachio P
2020-05-29	Modified	Cook J

Entities found in this pathway (2)

Input	UniProt Id	Input	UniProt Id
COL1A2	P08123	LAMA3	Q16787

19. Platelet Adhesion to exposed collagen (R-HSA-75892)

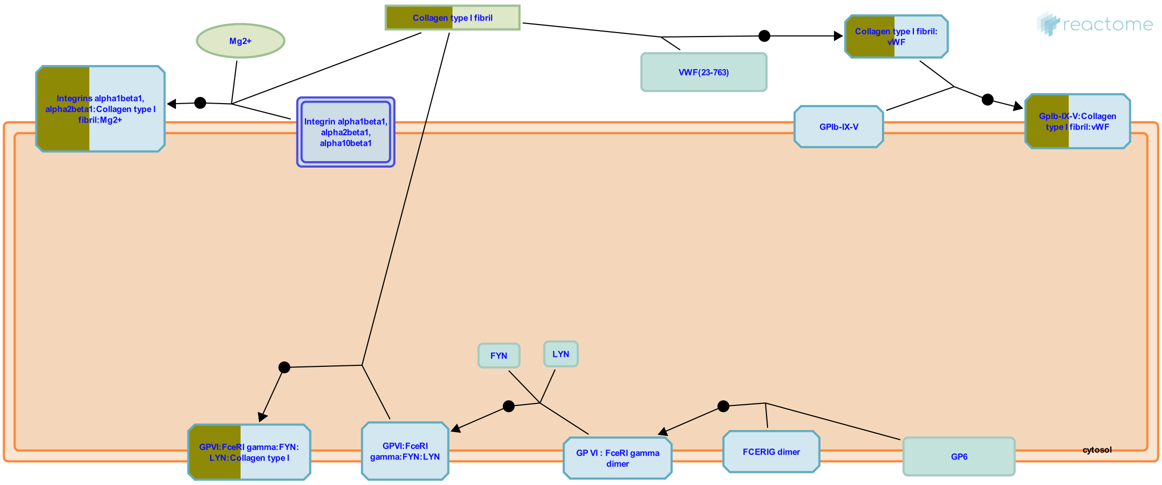

Initiation of platelet adhesion is the first step in the formation of the platelet plug. Circulating platelets are arrested and subsequently activated by exposed collagen and vWF. It is not entirely clear which type of collagen is responsible for adhesion and activation; collagen types I and III are abundant in vascular epithelia but several other types incluing IV are present (Farndale 2006). Several collagen binding proteins are expressed on platelets, including integrin alpha2 beta1, GPVI, and GPIV. Integrin alpha2 beta1, known on leukocytes as VLA-2, is the major platelet collagen receptor (Kunicki et al. 1988). It requires Mg2+ to interact with collagen and may require initiation mediated by the activation of integrin alphaIIb beta3 (van de Walle 2007). Binding occurs via the alpha2 subunit I domain to a collagen motif with the sequence Gly-Phe-Hyp-Gly-Glu-Arg (Emsley 2000). Binding of collagen to alpha2 beta1 generates intracellular signals that contribute to platelet activation. These facilitate the engagement of the lower-affinity collagen receptor, GPVI (Keely 1996), the key receptor involved in collagen-induced platelet activation. The GPVI receptor is a complex of the GPVI protein with a dimer of Fc epsilon R1 gamma (FceRI gamma). The Src family kinases Fyn and Lyn constitutively associate with the GPVI:FceRIgamma complex in platelets and initiate platelet activation through phosphorylation of the immunoreceptor tyrosine-based activation motif (ITAM) in FceRI gamma, leading to binding and activation of the tyrosine kinase Syk. Downstream of Syk, a series of adapter molecules and effectors lead to platelet activation. vWF protein is a polymeric structure of variable size. It is secreted in two directions, by the endothelium basolaterally and into the bloodstream. Shear-induced aggregation is achieved when vWF binds via its A1 domain to GPIb (part of GPIb-IX-V), and via its A3 domain mediating collagen binding to the subendothelium. The interaction between vWF and GPIb is regulated by shear force; an increase in the shear stress results in a corresponding increase in the affinity of vWF for GPIb.

References

Tsuji M, Ezumi Y, Arai M & Takayama H (1997). A novel association of Fc receptor gamma-chain with glycoprotein VI and their co-expression as a collagen receptor in human platelets. J Biol Chem, 272, 23528-31.

Miura Y, Takahashi T, Jung SM & Moroi M (2002). Analysis of the interaction of platelet collagen receptor glycoprotein VI (GPVI) with collagen. A dimeric form of GPVI, but not the monomeric form, shows affinity to fibrous collagen. J Biol Chem, 277, 46197-204.

Edit history

Date	Action	Author
2004-08-13	Authored	de Bono B
2004-09-25	Created	Farndale R, Pace NP, de Bono B
2020-05-29	Modified	Cook J

Entities found in this pathway (1)

Input	UniProt Id
COL1A2	P08123

20. CREB1 phosphorylation through the activation of Adenylate Cyclase (R-HSA-442720)

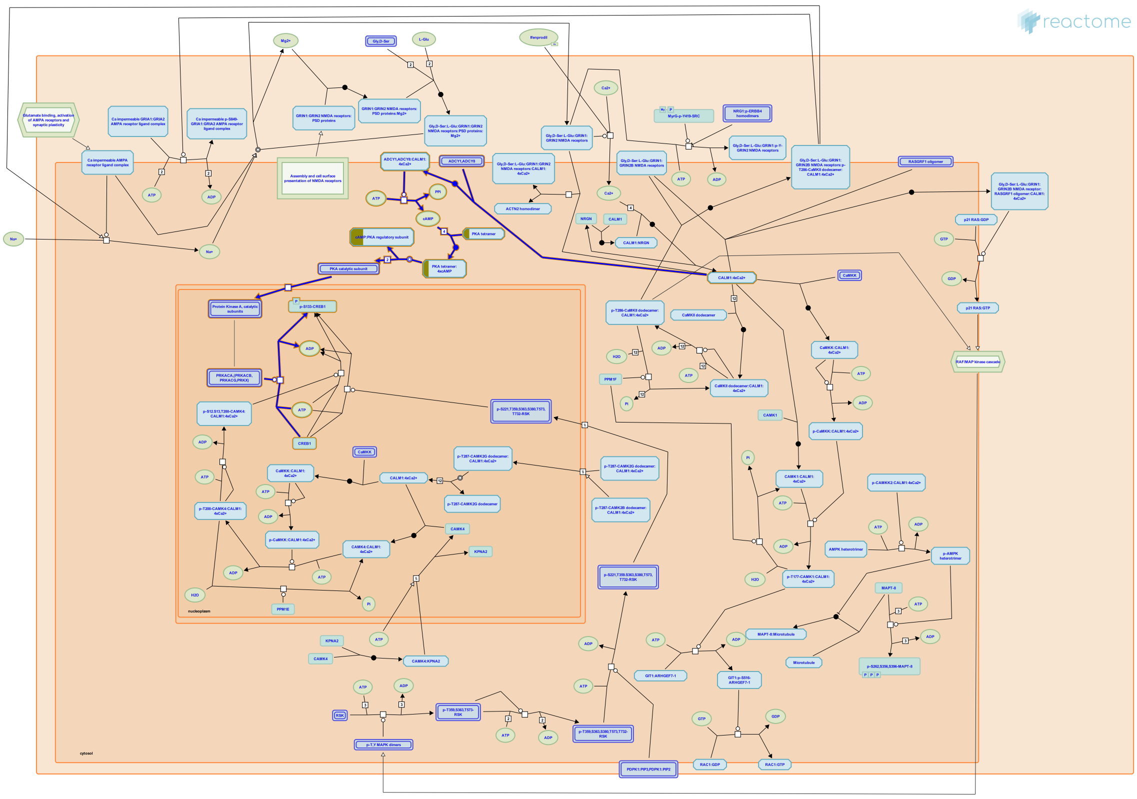

Cellular compartments: plasma membrane, cytosol, nucleoplasm.

Ca2+ influx through activated NMDA receptors in the post synaptic neurons activates adenylate cyclase-mediated signal transduction, leading to the activation of PKA and phosphorylation and activation of CREB1 induced transcription (Masada et al. 2012, Chetkovich et al. 1991, Chetkovich and Sweatt 1993

References

James MA, Lu Y, Liu Y, Vikis HG & You M (2009). RGS17, an overexpressed gene in human lung and prostate cancer, induces tumor cell proliferation through the cyclic AMP-PKA-CREB pathway. Cancer Res, 69, 2108-16.

Edit history

Date	Action	Author
2009-09-29	Created	Mahajan SS
2009-10-29	Authored	Mahajan SS
2009-11-18	Reviewed	Tukey D
2009-11-19	Edited	Gillespie ME
2018-10-10	Revised	Orlic-Milacic M
2018-11-02	Reviewed	Hansen KB, Yi F
2018-11-07	Edited	Orlic-Milacic M
2020-06-04	Modified	Cook J

Entities found in this pathway (1)

Input	UniProt Id
PRKAR2B	P31323

21. PKA activation (R-HSA-163615)

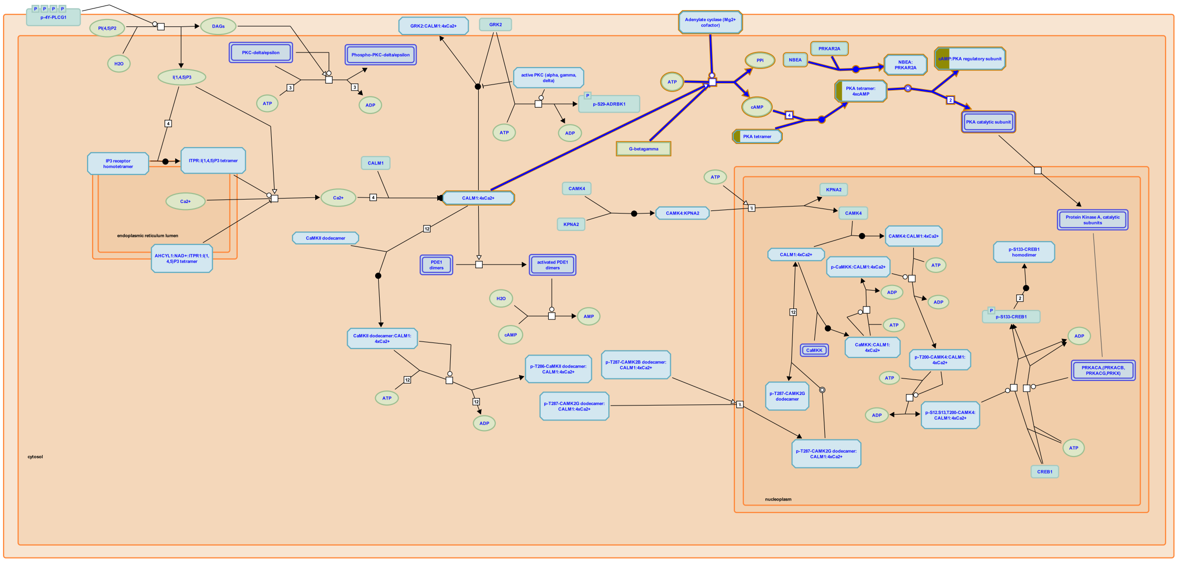

Cellular compartments: plasma membrane, cytosol.

A number of inactive tetrameric PKA holoenzymes are produced by the combination of homo- or heterodimers of the different regulatory subunits associated with two catalytic subunits. When cAMP binds to two specific binding sites on the regulatory subunits, these undergo a conformational change that causes the dissociation of a dimer of regulatory subunits bound to four cAMP from two monomeric, catalytically active PKA subunits.

References

Taylor SS, Buechler JA & Yonemoto W (1990). cAMP-dependent protein kinase: framework for a diverse family of regulatory enzymes. Annu Rev Biochem, 59, 971-1005.

Edit history

Date	Action	Author
2004-03-31	Authored	Le Novere N, Jassal B
2005-05-03	Created	Schmidt EE
2008-11-06	Edited	Jassal B
2008-11-06	Reviewed	Castagnoli L
2020-06-04	Modified	Cook J

Entities found in this pathway (1)

Input	UniProt Id
PRKAR2B	P31323

22. PKA activation in glucagon signalling (R-HSA-164378)

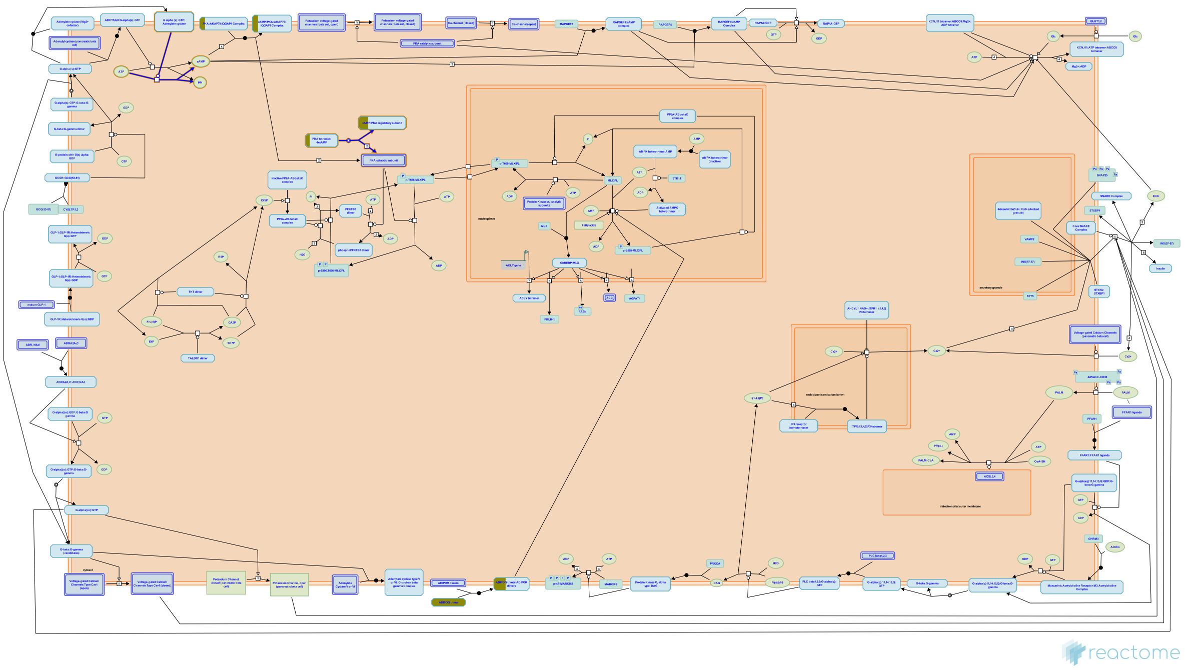

Cellular compartments: plasma membrane.

Adenylate cyclase catalyses the synthesis of cyclic AMP (cAMP) from ATP. In the absence of cAMP, protein kinase A (PKA) exists as inactive tetramers of two catalytic subunits and two regulatory subunits. cAMP binding to PKA tetramers causes them to dissociate and release their catalytic subunits as active monomers. Four isoforms of the regulatory subunit are known, that differ in their tissue specificity and functional characteristics, but the specific isoform activated in response to glucagon signaling has not yet been identified.

References

Edit history

Date	Action	Author
2005-05-19	Authored	Gopinathrao G, D'Eustachio P
2005-05-19	Created	Gopinathrao G
2016-11-08	Modified	Shorser S

Entities found in this pathway (1)

Input	UniProt Id
PRKAR2B	P31323

23. Crosslinking of collagen fibrils (R-HSA-2243919)

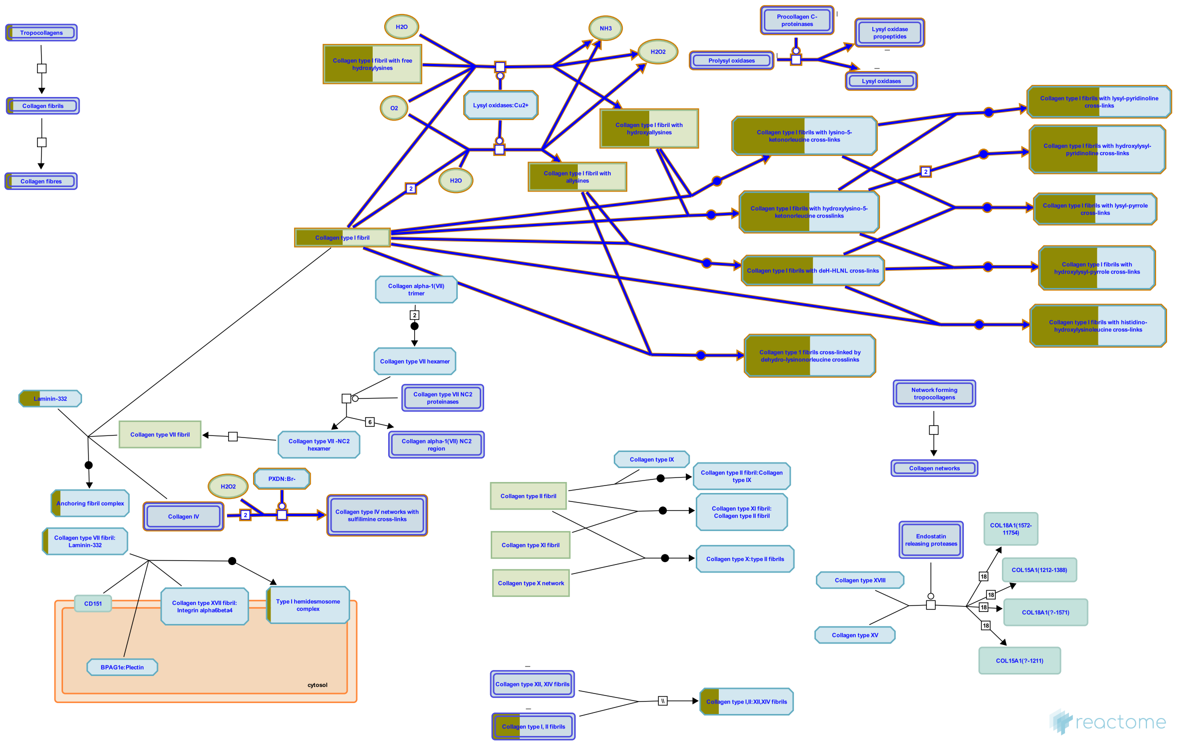

After removal of the N- and C-procollagen propeptides, fibrillar collagen molecules aggregate into microfibrillar arrays, stabilized by covalent intermolecular cross-links. These depend on the oxidative deamination of specific lysine or hydroxylysine residues in the telopeptide region by lysyl oxidase (LOX) with the subsequent spontaneous formation of covalent intermolecular cross-links (Pinnell & Martin 1968, Siegel et al. 1970, 1974, Maki 2009, Nishioka et al. 2012). Hydroxylysine is formed intracellularly by lysine hydroxylases (LH). There are different forms of LH responsible for hydroxylation of helical and telopeptide lysines (Royce & Barnes 1985, Knott et al.1997, Takaluoma et al. 2007, Myllyla 2007). The chemistry of the cross-links formed depends on whether lysines or hydroxylysines are present in the telopeptides (Barnes et al. 1974), which depends on the proportion of collagen lysines post-translationally converted to hydroxylysine by LH. The lysine pathway predominates in adult skin, cornea and sclera while the hydroxylysine pathway occurs primarily in bone, cartilage, ligament, tendons, embryonic skin and most connective tissues (Eyre 1987, Eyre & Wu 2005, Eyre et al. 2008). Oxidative deamination of lysine or hydroxylysine residues by LOX generates the allysine and hydroxyallysine aldehydes respectively. These can spontaneously react with either another aldehyde to form an aldol condensation product (intramolecular cross-link), or with an unmodified lysine or hydroxylysine residue to form intermolecular cross-links.

The pathway of cross-linking is regulated primarily by the hydroxylation pattern of telopeptide and triple-helix domain lysine residues. When lysine residues are the source of aldehydes formed by lysyl oxidase the allysine cross-linking pathway leads to the formation of aldimine cross-links (Eyre & Wu 2005). These are stable at physiological conditions but readily cleaved at acid pH or elevated temperature. When hydroxylysine residues are the source of aldehydes formed by lysyl oxidase the hydroxyallysine cross-linking pathway leads to the formation of more stable ketoimine cross-links.

Telopeptide lysine residues can be converted by LOX to allysine, which can react with a helical hydroxylysine residue forming the lysine aldehyde aldimine cross-link dehydro hydroxylysino norleucine (deHHLNL) (Bailey & Peach 1968, Eyre et al. 2008). If the telopeptide residue is hydroxylysine, the hydroxyallysine formed by LOX can react with a helical hydroxylysine forming the Schiff base, which spontaneously undergoes an Amadori rearrangement resulting in the ketoimine cross link hydroxylysino 5 ketonorleucine (HLKNL). This stable cross-link is formed in tissues where telopeptide residues are predominanly hydroxylated, such as foetal bone and cartilage, accounting for the relative insolubility of collagen from these tissues (Bailey et al. 1998). In bone, telopeptide hydroxyallysines can react with the epsilon-amino group of a helical lysine (Robins & Bailey 1975). The resulting Schiff base undergoes Amadori rearrangement to form lysino-hydroxynorleucine (LHNL). An alternative mechanism of maturation of ketoimine cross-links has been reported in cartilage leading to the formation of arginoline (Eyre et al. 2010).

These divalent crosslinks greatly diminish as connective tissues mature, due to further spontaneous reactions (Bailey & Shimokomaki 1971, Robins & Bailey 1973) with neighbouring peptides that result in tri- and tetrafunctional cross-links. In mature tissues collagen cross-links are predominantly trivalent. The most common are pyridinoline or 3-hydroxypyridinium cross-links, namely hydroxylysyl-pyridinoline (HL-Pyr) and lysyl-pyridinoline (L-Pyr) cross-links (Eyre 1987, Ogawa et al. 1982, Fujimoto et al. 1978). HL-Pyr is formed from three hydroxylysine residues, HLKNL plus a further hydroxyallysine. It predominates in highly hydroxylated collagens such as type II collagen in cartilage. L-Pyr is formed from two hydroxylysines and a lysine, LKNL plus a further hydroxyallysine, found mostly in calcified tissues (Bailey et al. 1998). Trivalent collagen cross-links can also form as pyrroles, either Lysyl-Pyrrole (L-Pyrrole) or hydroxylysyl-pyrrole (HL-Pyrrole), respectively formed when LKNL or HLKNL react with allysine (Scott et al. 1981, Kuypers et al. 1992). A further three-way crosslink can form when DeH-HLNL reacts with histidine to form histidino-hydroxylysinonorleucine (HHL), found in skin and cornea (Yamauchi et al. 1987, 1996). This can react with an additional lysine to form the tetrafunctional cross-link histidinohydroxymerodesmosine (Reiser et al. 1992, Yamauchi et al. 1996).

Another mechanism which could be involved in the cross-linking of collagen IV networks is the sulfilimine bond (Vanacore et al. 2009), catalyzed by peroxidasin, an enzyme found in basement membrane (Bhave 2012).

To improve clarity inter-chain cross-linking is represented here for Collagen type I only. Although the formation of each type of cross-link is represented here as an independent event, the partial and random nature of lysine hydroxylation and subsequent lysyl oxidation means that any combination of these cross-linking events could occur within the same collagen fibril .

References

Bailey AJ, Paul RG & Knott L (1998). Mechanisms of maturation and ageing of collagen. Mech Ageing Dev, 106, 1-56.

Edit history

Date	Action	Author
2012-04-30	Authored	Jupe S
2012-05-09	Created	Jupe S
2012-10-08	Reviewed	Kalamajski S, Raleigh S

Date	Action	Author
2012-11-12	Edited	Jupe S
2012-11-19	Reviewed	Ricard-Blum S
2020-05-29	Modified	Cook J

Entities found in this pathway (1)

Input	UniProt Id
COL1A2	P08123

24. Interleukin-4 and Interleukin-13 signaling (R-HSA-6785807)

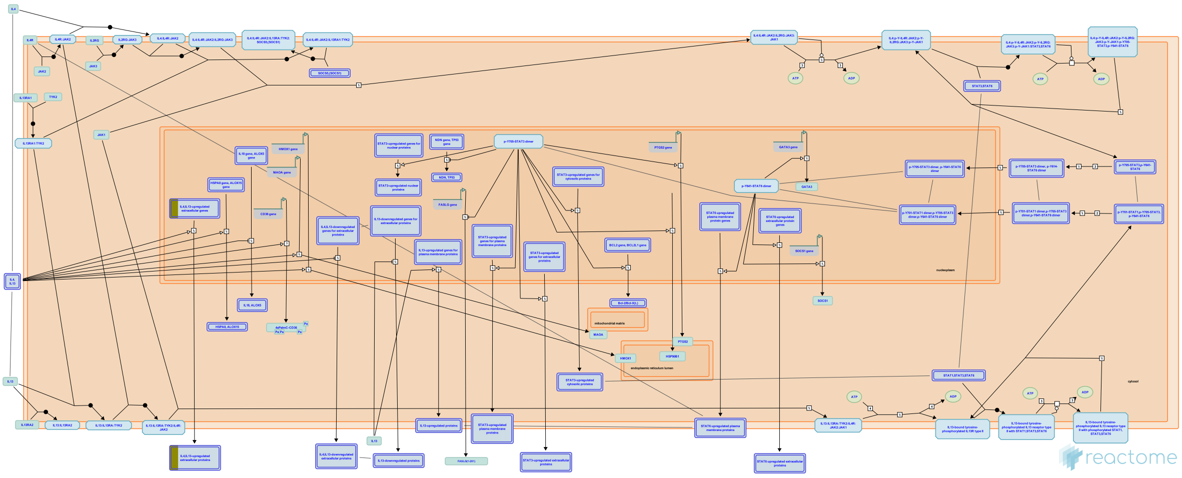

Interleukin-4 (IL4) is a principal regulatory cytokine during the immune response, crucially important in allergy and asthma (Nelms et al. 1999). When resting T cells are antigen-activated and expand in response to Interleukin-2 (IL2), they can differentiate as Type 1 (Th1) or Type 2 (Th2) T helper cells. The outcome is influenced by IL4. Th2 cells secrete IL4, which both stimulates Th2 in an autocrine fashion and acts as a potent B cell growth factor to promote humoral immunity (Nelms et al. 1999).

Interleukin-13 (IL13) is an immunoregulatory cytokine secreted predominantly by activated Th2 cells. It is a key mediator in the pathogenesis of allergic inflammation. IL13 shares many functional properties with IL4, stemming from the fact that they share a common receptor subunit. IL13 receptors are expressed on human B cells, basophils, eosinophils, mast cells, endothelial cells, fibroblasts, monocytes, macrophages, respiratory epithelial cells, and smooth muscle cells, but unlike IL4, not T cells. Thus IL13 does not appear to be important in the initial differentiation of CD4 T cells into Th2 cells, rather it is important in the effector phase of allergic inflammation (Hershey et al. 2003).

IL4 and IL13 induce “alternative activation” of macrophages, inducing an anti-inflammatory phenotype by signaling through IL4R alpha in a STAT6 dependent manner. This signaling plays an important role in the Th2 response, mediating anti-parasitic effects and aiding wound healing (Gordon & Martinez 2010, Loke et al. 2002)

There are two types of IL4 receptor complex (Andrews et al. 2006). Type I IL4R (IL4R1) is predominantly expressed on the surface of hematopoietic cells and consists of IL4R and IL2RG, the common gamma chain. Type II IL4R (IL4R2) is predominantly expressed on the surface of nonhematopoietic cells, it consists of IL4R and IL13RA1 and is also the type II receptor for IL13. (Obiri et al. 1995, Aman et al. 1996, Hilton et al. 1996, Miloux et al. 1997, Zhang et al. 1997). The second receptor for IL13 consists of IL4R and Interleukin-13 receptor alpha 2 (IL13RA2), sometimes called Interleukin-13 binding protein (IL13BP). It has a high affinity receptor for IL13 (Kd = 250 pmol/L) but is not sufficient to render cells responsive to IL13, even in the presence of IL4R (Donaldson et al. 1998). It is reported to exist in soluble form (Zhang et al. 1997) and when overexpressed reduces JAK-STAT signaling (Kawakami et al. 2001). It's function may be to prevent IL13 signalling via the functional IL4R:IL13RA1 receptor. IL13RA2 is overexpressed and enhances cell invasion in some human cancers (Joshi & Puri 2012).

The first step in the formation of IL4R1 (IL4:IL4R:IL2RB) is the binding of IL4 with IL4R (Hoffman et al. 1995, Shen et al. 1996, Hage et al. 1999). This is also the first step in formation of IL4R2 (IL4:IL4R:IL13RA1). After the initial binding of IL4 and IL4R, IL2RB binds (LaPorte et al. 2008), to form IL4R1. Alternatively, IL13RA1 binds, forming IL4R2. In contrast, the type II IL13 complex (IL13R2) forms with IL13 first binding to IL13RA1 followed by recruitment of IL4R (Wang et al. 2009).

Crystal structures of the IL4:IL4R:IL2RG, IL4:IL4R:IL13RA1 and IL13:IL4R:IL13RA1 complexes have been determined (LaPorte et al. 2008). Consistent with these structures, in monocytes IL4R is tyrosine phosphorylated in response to both IL4 and IL13 (Roy et al. 2002, Gordon & Martinez 2010) while IL13RA1 phosphorylation is induced only by IL13 (Roy et al. 2002, LaPorte et al. 2008) and IL2RG phosphorylation is induced only by IL4 (Roy et al. 2002).

Both IL4 receptor complexes signal through Jak/STAT cascades. IL4R is constitutively-associated with JAK2 (Roy et al. 2002) and associates with JAK1 following binding of IL4 (Yin et al. 1994) or IL13 (Roy et al. 2002). IL2RG constitutively associates with JAK3 (Boussiotis et al. 1994, Russell et al. 1994). IL13RA1 constitutively associates with TYK2 (Umeshita-Suyama et al. 2000, Roy et al. 2002, LaPorte et al. 2008, Bhattacharjee et al. 2013).

IL4 binding to IL4R1 leads to phosphorylation of JAK1 (but not JAK2) and STAT6 activation (Takeda et al. 1994, Ratthe et al. 2007, Bhattacharjee et al. 2013).

IL13 binding increases activating tyrosine-99 phosphorylation of IL13RA1 but not that of IL2RG. IL4 binding to IL2RG leads to its tyrosine phosphorylation (Roy et al. 2002). IL13 binding to IL4R2 leads to TYK2 and JAK2 (but not JAK1) phosphorylation (Roy & Cathcart 1998, Roy et al. 2002).

Phosphorylated TYK2 binds and phosphorylates STAT6 and possibly STAT1 (Bhattacharjee et al. 2013).

A second mechanism of signal transduction activated by IL4 and IL13 leads to the insulin receptor substrate (IRS) family (Kelly-Welch et al. 2003). IL4R1 associates with insulin receptor substrate 2 and activates the PI3K/Akt and Ras/MEK/Erk pathways involved in cell proliferation, survival and translational control. IL4R2 does not associate with insulin receptor substrate 2 and consequently the PI3K/Akt and Ras/MEK/Erk pathways are not activated (Busch-Dienstfertig & González-Rodríguez 2013).

References

Nelms K, Keegan AD, Zamorano J, Ryan JJ & Paul WE (1999). The IL-4 receptor: signaling mechanisms and biologic functions. Annu. Rev. Immunol., 17, 701-38.

Hershey GK (2003). IL-13 receptors and signaling pathways: an evolving web. J. Allergy Clin. Immunol., 111, 677-90; quiz 691.

Edit history

Date	Action	Author
2015-07-01	Authored	Jupe S
2015-07-01	Created	Jupe S
2016-09-02	Edited	Jupe S
2016-09-02	Reviewed	Leibovich SJ

Date	Action	Author
2020-06-04	Modified	Cook J

Entities found in this pathway (1)

Input	UniProt Id
COL1A2	P08123

Input	Ensembl Id
COL1A2	ENSG00000164692

25. PKA-mediated phosphorylation of CREB (R-HSA-111931)

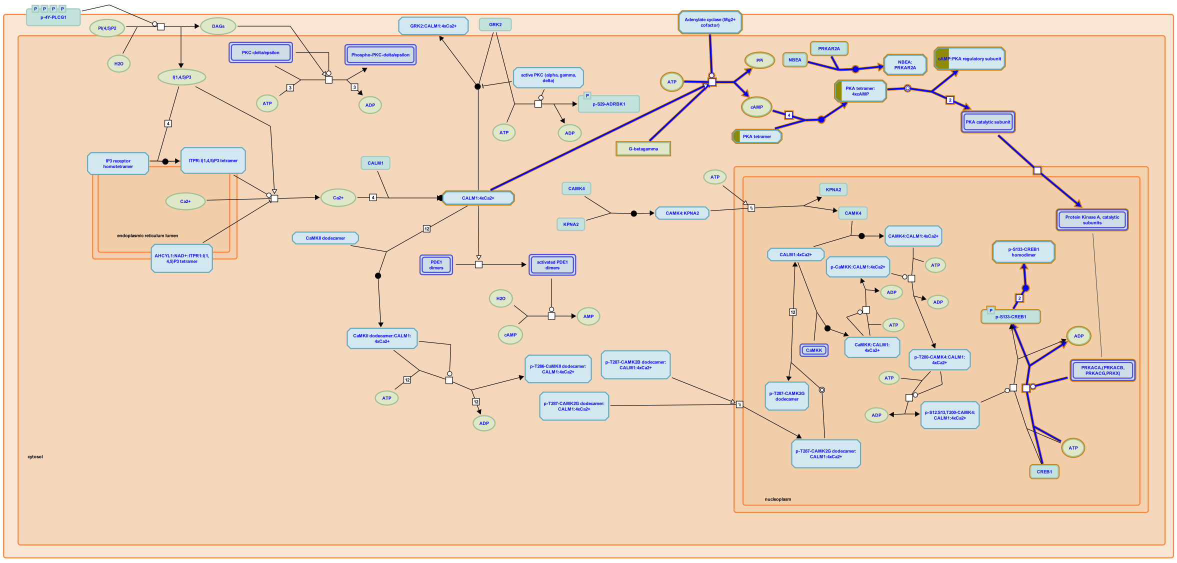

reactome

Cellular compartments: plasma membrane, nucleoplasm, cytosol.

Cyclic adenosine 3',5'-monophosphate (cAMP) induces gene transcription through activation of cAMP-dependent protein kinase (PKA), and subsequent phosphorylation of the transcription factor cAMP response element-binding protein, CREB, at serine-133.

References

Johannessen M, Delghandi MP & Moens U (2004). What turns CREB on?. Cell Signal, 16, 1211-27.

Delghandi MP, Johannessen M & Moens U (2005). The cAMP signalling pathway activates CREB through PKA, p38 and MSK1 in NIH 3T3 cells. Cell Signal, 17, 1343-51.

Edit history

Date	Action	Author
2004-03-25	Created	Schmidt EE
2004-03-31	Authored	Le Novere N, Jassal B
2008-11-06	Edited	Jassal B
2008-11-06	Reviewed	Castagnoli L
2012-11-26	Modified	D'Eustachio P

Entities found in this pathway (1)

Input	UniProt Id
PRKAR2B	P31323

6. Identifiers found

Below is a list of the input identifiers that have been found or mapped to an equivalent element in Reactome, classified by resource.

Entities (13)

Input	UniProt Id	Input	UniProt Id	Input	UniProt Id
ABCF1	Q8NE71	ABHD14B	Q96IU4	ALDH18A1	P54886-1, P54886-2
COL1A2	P08123	EIF4A2	Q15848	EMILIN1	Q9Y6C2
GDI1	P31150, P50395	HMBS	P08397	KRT1	P04264
LAMA3	Q16787	NARS	O43776	PRKAR2B	P31323
SUCLA2	Q9P2R7				

Input	Ensembl Id
COL1A2	ENSG00000164692

7. Identifiers not found

These 3 identifiers were not found neither mapped to any entity in Reactome.

ABI3BP	BZW2	MYOC
