# Supplementary figures and images for: Human amniotic epithelial cell transplantation improves scar remodeling in a rabbit model of acute vocal fold injury: a pilot study
Source: Stem Cell Res Ther. 2022 Jan 25;13:31. doi: 10.1186/s13287-022-02701-w (PMC8787902; doi:10.1186/s13287-022-02701-w)

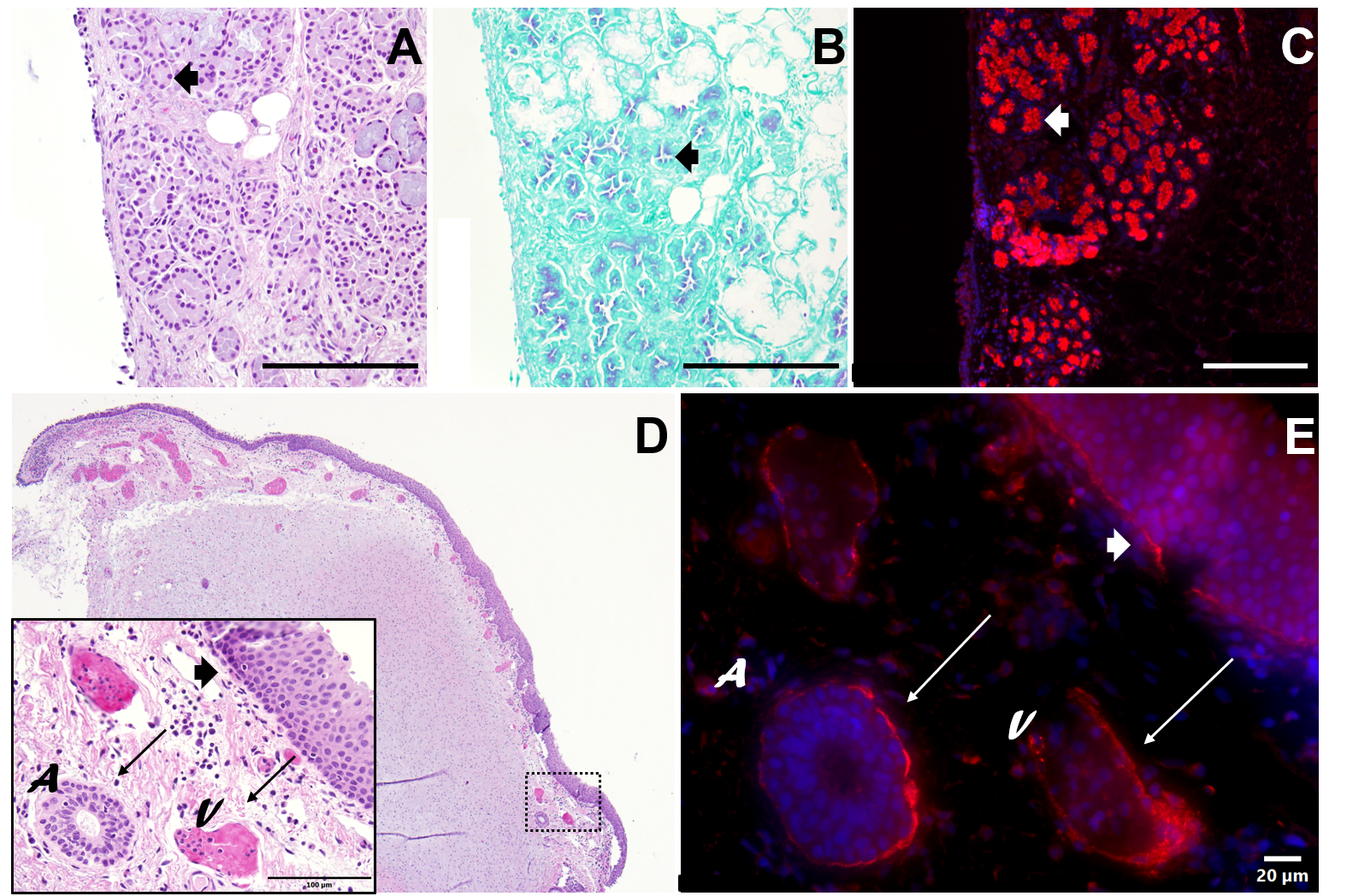

Supplement: Supplementary file 2 — Additional file 2: Fig. S1. Immunofluorescence for myocilin in submucosal gland adjacent to the vocal fold in a rabbit larynx and in human vocal fold. Representative images of the glandular epithelium adjacent to the vocal fold show its general histology with H&E staining (A), the presence of muco-substances intracellularly with PAS staining (B magenta color) and the expression of myocilin in the glandular secreting cells with immunofluorescence (C). In the human vocal fold (D, E), the myocilin-positive sites are the adventitia of arterioles (A) and venules (V) (thin arrows) and the basal membrane (thick arrows). Scale Bars: 200 µm (A–C), 100 µm (D insert), and 20 µm (E). [file 13287_2022_2701_MOESM2_ESM.tif]
